# Eosinophil function in adipose tissue is regulated by Krüppel-like factor 3 (KLF3)

**DOI:** 10.1038/s41467-020-16758-9

**Published:** 2020-06-10

**Authors:** Alexander J. Knights, Emily J. Vohralik, Peter J. Houweling, Elizabeth S. Stout, Laura J. Norton, Stephanie J. Alexopoulos, Jinfen. J. Yik, Hanapi Mat Jusoh, Ellen M. Olzomer, Kim S. Bell-Anderson, Kathryn N. North, Kyle L. Hoehn, Merlin Crossley, Kate G. R. Quinlan

**Affiliations:** 1 School of Biotechnology and Biomolecular Sciences, UNSW Sydney, Sydney, NSW 2052 Australia; 2 Murdoch Children’s Research Institute, The Royal Children’s Hospital, Melbourne, VIC 3052 Australia; 3 Department of Paediatrics, University of Melbourne, Melbourne, VIC 3052 Australia; 4 Charles Perkins Centre, School of Life and Environmental Sciences, University of Sydney, Sydney, NSW 2006 Australia

**Keywords:** Gene regulation, Eosinophils, Obesity

## Abstract

The conversion of white adipocytes to thermogenic beige adipocytes represents a potential mechanism to treat obesity and related metabolic disorders. However, the mechanisms involved in converting white to beige adipose tissue remain incompletely understood. Here we show profound beiging in a genetic mouse model lacking the transcriptional repressor Krüppel-like factor 3 (KLF3). Bone marrow transplants from these animals confer the beige phenotype on wild type recipients. Analysis of the cellular and molecular changes reveal an accumulation of eosinophils in adipose tissue. We examine the transcriptomic profile of adipose-resident eosinophils and posit that KLF3 regulates adipose tissue function via transcriptional control of secreted molecules linked to beiging. Furthermore, we provide evidence that eosinophils may directly act on adipocytes to drive beiging and highlight the critical role of these little-understood immune cells in thermogenesis.

## Abstract (web summary)

Immune cells are important regulators of adipose tissue function, including adaptive thermogenesis. Here the authors show that mice with Krüppel-like factor 3 (KLF3) deficiency in bone marrow-derived cells have increased adipose tissue beiging which may at least in part be due to altered eosinophil paracrine signaling.

## Introduction

White adipose tissue (AT), typically regarded as an energy storage site, can acquire the thermogenic properties of brown AT to become ‘beige’ and drive energy expenditure^1,2^. The discovery that AT can be activated in this way may be important in combatting obesity and associated cardiometabolic disorders.

It is now apparent that resident immune cells, particularly mediators of type 2 immunity, are involved in beige AT activation and energy expenditure. Eosinophils have been implicated in beiging^3–5^ but the underlying cellular and molecular mechanisms orchestrating their contributions remain incompletely understood. Obese mice have fewer eosinophils in their AT than lean counterparts, and the ΔdblGATA transgenic mouse, which lacks eosinophils altogether, displays exaggerated weight gain on a high-calorie diet^4^. Using the ΔdblGATA model, it has also been proposed that AT eosinophils facilitate adipocyte maturation to ameliorate diabetic complications of diet-induced obesity^6^. Conversely, mice genetically engineered to overexpress interleukin 5 (IL-5) have supraphysiological levels of eosinophils and are protected from diet-induced obesity^4^. However, a recent study reported that artificially increasing eosinophils in AT with recombinant IL-5 did not confer the expected metabolic benefits, leading the authors to suggest that the functional activities of eosinophils may be more crucial than their abundance^7^. Furthermore, the principal contribution of macrophages to AT energy expenditure—their production of catecholamines^8^—is currently under debate, with several groups finding that rather than synthesising catecholamines, macrophages are instead responsible for catecholamine uptake and degradation^9–11^. Thus, interest in the key cellular and molecular drivers of beiging remains intense.

We have previously shown that mice lacking the transcriptional repressor Krüppel-like factor 3 (KLF3) have reduced adiposity and are protected from diet-induced obesity^12,13^. Here we report enhanced beige AT activation in *Klf3*^*−/−*^ mice and that bone marrow (BM) transplants from these mice confer the lean, beige phenotype on recipients. In the absence of KLF3, AT-resident eosinophils are more abundant and exhibit significant deregulation of important secreted molecules, including meteorin-like and IL-33, both of which influence beiging^5,14–16^. We also report that co-culture of eosinophils with primary adipocytes increases thermogenic gene expression. These findings identify KLF3 as an important regulator of AT eosinophil gene expression and function, advancing our understanding of how these little-understood immune cells may lead to improved strategies for therapeutically driving energy expenditure.

## Results

### Reduced adiposity and enhanced beiging in *Klf3*^−/−^ mice

**Fig. 1:**
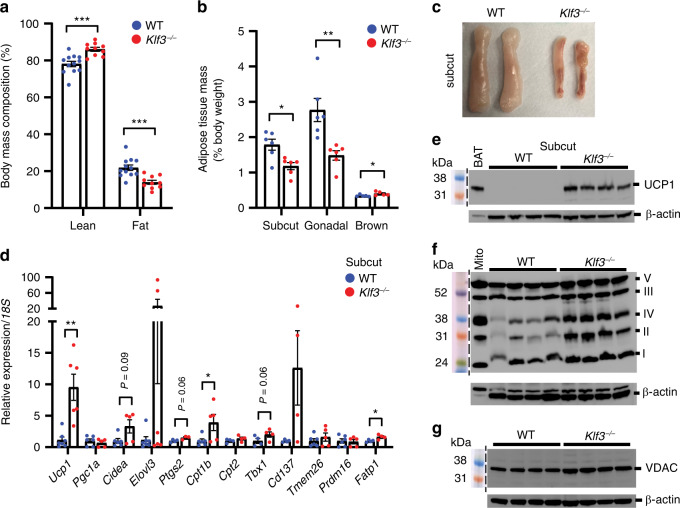
Reduced adiposity and enhanced beiging in *Klf3*^*−/−*^ mice. **a** Lean and fat body mass composition (%) of WT (*n* = 12) and *Klf3*^*−/−*^ (*n* = 10) mice were assessed by EchoMRI analysis. **b** Weights of WT and *Klf3*^*−/−*^ AT depots were recorded as a percentage of total body weight (*n* = 6 mice for subcut and gonadal AT; *n* = 5 mice for brown AT). **c** Representative macroscopic images of WT and *Klf3*^*−/−*^ subcut AT pads showing relative size and complexion. **d** mRNA levels of thermogenic genes were assessed by qPCR in WT and *Klf3*^*−/−*^ subcut AT (*n* = 4–8 mice). Relative expression was normalised to *18S* rRNA levels and the mean WT value for each gene was set to 1. **e** UCP1 protein expression was measured in WT and *Klf3*^*−/−*^ subcut AT by western blotting (*n* = 4 mice). 25 μg of whole-cell extract (WCE) was loaded alongside a Rainbow molecular weight marker. Nitrocellulose membranes were probed with anti-UCP1 antibody overnight. Diluted brown adipose tissue (BAT) extract included as a positive control. **f** Expression of mitochondrial oxphos proteins in WT and *Klf3*^*−/−*^ subcut AT WCE was assessed by western blotting (*n* = 4 mice). 25 μg of extract was loaded alongside rat heart mitochondria extract (Mito; positive control), and PVDF membranes were blocked overnight before being probed with the Total OXPHOS Rodent WB Antibody Cocktail for 2 h. Mitochondrial complexes are labelled by their Roman numerals (I–V). **g** VDAC protein expression was measured in WT and *Klf3*^*−/−*^ subcut AT (*n* = 4 mice) by western blotting. 20 μg of WCE was loaded and nitrocellulose membranes were probed overnight with anti-VDAC antibody. For **e**–**g** β-actin was included as a loading control. For **a**, **b** and **d**, error bars represent means ± SEM and one-sided non-parametric Mann–Whitney *U* tests were performed where *P < 0.05, **P < 0.01, ***P < 0.001. Source data are provided as a Source data file. kDa, kilodaltons. See also Supplementary Figs. 1 and 2.

Disruption of the gene encoding the transcriptional repressor KLF3 results in mice that are smaller than their wild type (WT) littermates with less white AT^13^ (Supplementary Fig. 1a, b), and confers protection from diet-induced obesity^12^. Body mass composition analysis of chow-fed WT and *Klf3*^*−/−*^ animals housed at room temperature (22 °C) showed that *Klf3*^*−/−*^ mice exhibit reduced total fat mass compared to WT littermates (Fig. 1a and Supplementary Fig. 1a), in addition to differences in lean body mass, which constitute their reduced body weight (Supplementary Fig. 1b). This is reflected in the reduced size of white AT depots in *Klf3*^*−/−*^ mice seen previously^12^ (Fig. 1b and Supplementary Fig. 1c). Visual examination of subcutaneous (subcut) AT depots, the depots most prone to beiging^17^, revealed a browner complexion and smaller size in *Klf3*^*−/−*^ mice (Fig. 1c). Furthermore, H&E staining revealed that in the absence of KLF3, adipose cellular architecture is notably altered, with enrichment of multilocular adipocytes evident that was not seen in the subcut AT of WT mice (Supplementary Fig. 2a, b). These observations also confirm the previous finding that *Klf3*^*−/−*^ mice have smaller-sized adipocytes^12,13^. Given that thermogenic energy expenditure via activation of beige AT may influence adiposity^18–20^, we examined the expression of archetypal thermogenic genes. We observed upregulation of numerous thermogenic genes in the subcut AT of *Klf3*^*−/−*^ mice – most notably *Ucp1*, *Cpt1b*, *Fatp1* and the beige-specific marker^21^ *Tbx1* (Fig. 1d). We next investigated the levels of mitochondrial proteins by Western blotting of whole-cell extracts (WCE) from WT and *Klf3*^*−/−*^ subcut AT. Uncoupling protein 1 (UCP1) protein levels were higher in the subcut AT of *Klf3*^*−/−*^ mice (Fig. 1e), as were mitochondrial oxidative phosphorylation (oxphos) complexes I–V (Fig. 1f). We also observed increased levels of the mitochondrial outer membrane protein voltage-dependent anion channel (VDAC) in *Klf3*^*−/−*^ subcut AT (Fig. 1g), suggesting higher mitochondrial number. Levels of multiple thermogenic genes were also increased in the gonadal AT of *Klf3*^*−/−*^ mice (Supplementary Fig. 1d), as were UCP1 and mitochondrial oxphos proteins (Supplementary Fig. 1e, f). Several genes were modestly increased in interscapular brown AT of *Klf3*^*−/−*^ mice while beige-specific markers^1^ *Tbx1*, *Tmem26* and *Cd137* were undetectable (Supplementary Fig. 1g). UCP1 protein content was mildly decreased in *Klf3*^*−/−*^ brown AT (Supplementary Fig. 1h, i). While this suggests that brown AT is unlikely to play a major role in the thermogenic phenotype of *Klf3*^*−/−*^ mice, we cannot wholly rule out its contribution given the existence of UCP1-independent thermogenic mechanisms in beige and brown fat^22–25^. Together, these results show that *Klf3*^*−/−*^ mice exhibit reduced fat mass that may result from enhanced AT beiging, as demonstrated by the widespread de-repression of thermogenic genes and mitochondrial proteins.

### KLF3 deficiency enhances the thermogenic response

**Fig. 2:**
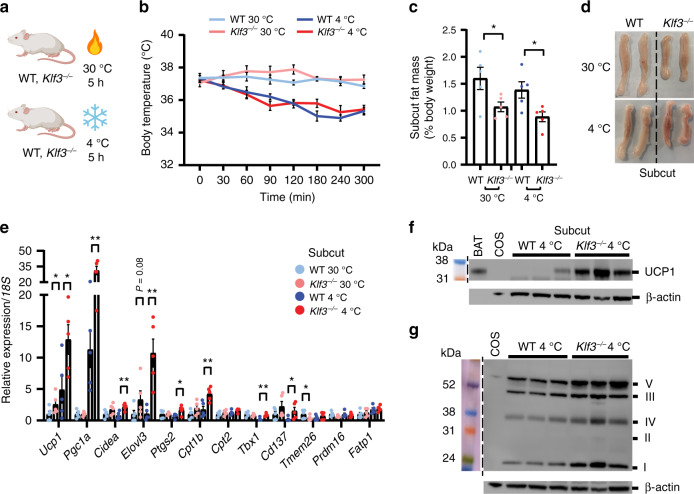
KLF3 deficiency enhances the thermogenic response. **a** WT and *Klf3*^*−/−*^ mice were acutely housed at 30 °C or 4 °C (cartoon created with BioRender.com) and **b** body temperature was monitored by rectal probing over a 5 h period (*n* = 5 mice). **c** Weights of WT and *Klf3*^*−/−*^ subcut AT from mice housed at 30 °C or 4 °C were recorded as relative to body weight (*n* = 5 mice.) **d** Representative macroscopic images of subcut AT from WT and *Klf3*^*−/−*^ mice housed at 30 °C or 4 °C, showing size and complexion. **e** mRNA levels of thermogenic genes were assessed by qPCR in WT and *Klf3*^*−/−*^ subcut AT (*n* = 5 mice). Relative expression was normalised to *18S* rRNA levels and the WT 30 °C value for each gene was set to 1. **f** UCP1 protein expression was measured in subcut AT from WT and *Klf3*^*−/−*^ mice housed at 4 °C by western blotting (*n* = 3 mice). 25 μg of WCE was loaded alongside a Rainbow molecular weight marker. Nitrocellulose membranes were probed with anti-UCP1 antibody overnight. β-actin was included as a loading control and diluted brown AT (BAT) extract included as a positive control. **g** Expression of mitochondrial oxphos proteins in subcut AT from WT and *Klf3*^*−/−*^ mice housed at 4 °C was assessed by western blotting (*n* = 3 mice). 25 μg of extract was loaded alongside rat heart mitochondria extract (Mito; positive control), and PVDF membranes were blocked overnight before being probed with the Total OXPHOS Rodent WB Antibody Cocktail for 2 h. β-actin was including as a loading control. Mitochondrial complexes are labelled by their Roman numerals (I–V). Extracts from COS cells were included as negative control lanes. For **b** two-way ANOVA followed by post-hoc Tukey testing was performed to assess statistical significance. For **c**, **e**, error bars represent means ± SEM and one-sided non-parametric Mann–Whitney *U* tests were performed where *P < 0.05, **P < 0.01. Source data are provided as a Source data file. kDa, kilodaltons; COS, COS-7 cells. See also Supplementary Fig. 3.

Cold exposure is a recognised means of promoting adaptive thermogenesis in AT^26–28^. To study whether the absence of KLF3 enhances beige AT activation and influences body temperature both in the cold and at thermoneutrality, we acutely exposed WT and *Klf3*^*−/−*^ mice to ambient temperatures of 4 °C or 30 °C (Fig. 2a). No statistically significant difference was seen in the body temperatures of WT and *Klf3*^*−/−*^ mice during cold exposure (Fig. 2b). As expected, there was little disparity in body temperature between WT and *Klf3*^*−/−*^ mice housed at 30 °C. Subcut (Fig. 2c and Supplementary Fig. 3a) and gonadal AT (Supplementary Fig. 3b, c) from *Klf3*^*−/−*^ mice were smaller than WT depots under both conditions, and interestingly, interscapular brown AT was smaller in *Klf3*^*−/−*^ mice acutely exposed to 4 °C than in WT mice at 4 °C or in *Klf3*^*−/−*^ mice at thermoneutrality (Supplementary Figs. 3d, e). Visual inspection of subcut AT depots from mice housed at 30 °C and 4 °C showed that *Klf3*^*−/−*^ mice exhibited distinct, enhanced evidence of browning at 4 °C compared to WT mice, and this disparity was retained at 30 °C though to a lesser extent (Fig. 2d). This striking change in macroscopic appearance was surprising, given the short period of cold exposure (5 h), and may in part be strain-dependent and due to the elevated levels of basal thermogenesis in *Klf3*^*−/−*^ mice. To examine beige AT activation at the transcriptional level, we assessed expression of thermogenic genes in subcut AT from mice housed at 30 °C and 4 °C. At 4 °C, we found that multiple genes were increased in the absence of KLF3, including *Ucp1*, *Pgc1a, Cidea*, *Elovl3* and *Cpt1b* (Fig. 2e), with similar deregulation seen in gonadal AT (Supplementary Fig. 3f) and to a lesser extent, brown AT (Supplementary Fig. 3g). *Ucp1* mRNA expression was elevated in subcut AT from *Klf3*^*−/−*^ mice compared to WT mice housed at 30 °C. Western blotting of subcut UCP1 and mitochondrial oxphos complexes confirmed upregulation at the protein level at 4 °C (Fig. 2f, g). Thus, under thermal stress at 4 °C, activation of beige AT is amplified in *Klf3*^*−/−*^ mice. At thermoneutrality, KLF3 deficiency still augments beige AT activation, suggesting that the effects seen are not solely due to cold temperature, but that in this model beiging of white AT can occur independently of thermal stress.

### Reduced weight gain in WT mice transplanted with Klf3^−/−^ BM

**Fig. 3:**
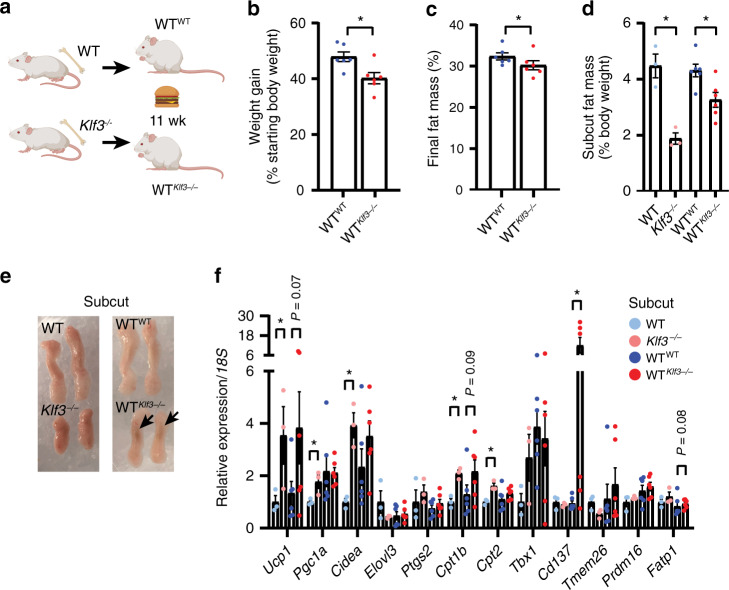
Reduced weight gain in WT mice transplanted with *Klf3*^*−/−*^ BM. **a** WT mice were irradiated and transplanted with bone marrow from WT (WT^WT^) or *Klf3*^*−/−*^ (WT^*Klf3−/−*^) donors then fed a high-fat, high-sugar (Western) diet for 11 weeks (wk) (cartoon created with BioRender.com). **b** Body weight was monitored throughout the Western diet study duration (weeks 2–13), and the total weight gained was calculated as a percentage of starting body weight at commencement of Western diet administration (*n* = 6 mice). **c** Fat mass composition (%) was assessed using EchoMRI after 11 weeks of Western diet (*n* = 6 mice). **d** Weights of subcut AT (relative to body weight) from WT, *Klf3*^*−/−*^ (*n* = 3), WT^WT^ and WT^*Klf3−/−*^ (*n* = 6) mice fed a Western diet for 11 weeks. **e** Representative macroscopic images of subcut AT pads from mice of each condition, showing size and complexion. Arrows point to sites of enriched brown appearance. **f** mRNA levels of thermogenic genes in the subcut AT of WT, *Klf3*^*−/−*^ (*n* = 3), WT^WT^ and WT^*Klf3−/−*^ (*n* = 6) mice were assessed by qPCR. Relative expression was normalised to levels of *18S* rRNA and the mean WT value set to 1 for each target. For **b**–**d** and **f**, error bars represent means ± SEM and one-sided non-parametric Mann–Whitney *U* tests were performed where *P < 0.05. Source data are provided as a Source data file. See also Supplementary Figs. 4, 5.

We have previously shown that mice lacking KLF3 are protected from obesity when fed a high-fat diet, with no difference exhibited in food intake on either a chow or high-fat diet compared to WT mice^12^. While the mechanisms underlying had remained unclear, evidence of enhanced beiging in *Klf3*^*−/−*^ mice now provides a possible explanation. The role of haematopoietic cells in AT function and thermogenesis is now widely appreciated, and KLF3 is both active in haematopoietic lineages^29–33^ and highly expressed in a range of haematopoietic cell types according to the Immgen^34^ and Haemopedia^35,36^ databases. We thus focused on a possible role for KLF3 in BM haematopoietic cells that could give rise to cells occupying the AT SVF niche and regulate adipose function. We irradiated WT mice and transplanted them with BM from WT or *Klf3*^*−/−*^ mice to produce WT^WT^ and WT^*Klf3−/−*^ chimeras (Fig. 3a). Chimeric mice and a control cohort of untransplanted WT and *Klf3*^*−/−*^ animals were fed a high-fat, high-sugar Western diet for 11 weeks in order to investigate resistance to diet-induced obesity and adiposity. Successful haematopoietic reconstitution was assessed by genotyping tail biopsies (recipient and donor cells) and BM (recipient cells only) (Supplementary Fig. 4a). Prior to commencing the Western diet regimen, WT^*Klf3−/−*^ chimeras were heavier than WT^WT^ counterparts, then over the 11-week Western diet period, WT^*Klf3−/−*^ chimeras gained modestly but significantly less weight than WT^WT^ counterparts (Fig. 3b and Supplementary Fig. 4b, c) and had less body fat (Fig. 3c). Importantly, WT^*Klf3−/−*^ chimeras gained less subcut AT mass compared to WT^WT^ animals (Fig. 3d and Supplementary Fig. 4d) and more prominent browning in WT^*Klf3−/−*^ subcut AT was detected by visual inspection (Fig. 3e). No such differences were observed in the weights or appearance of gonadal or brown AT (Supplementary Fig. 4e–i). The expression of thermogenic genes in the subcut AT of chimeric mice was tested and *Ucp1*, *Cpt1b* and *Cd137* were found to be upregulated in animals that received *Klf3*^*−/−*^ BM compared to mice that received WT BM (Fig. 3f). Several genes were also upregulated in WT^*Klf3−/−*^ gonadal and brown AT (Supplementary Fig. 4j, k). We performed biochemical analysis on the peripheral blood of transplanted mice and showed that plasma triglycerides were significantly lower in WT^*Klf3−/−*^ mice compared to WT^WT^, while no difference existed in total cholesterol (Supplementary Fig. 5a, b). We also detected reduced hepatic triglycerides in untransplanted KLF3-deficient mice (Supplementary Fig. 5c), and observed no difference in liver cholesterol levels (Supplementary Fig. 5d). Together these results demonstrate that transplantation of irradiated WT mice with KLF3-deficient BM was sufficient to confer metabolic benefits on recipient mice. This suggests that haematopoietic cells may be instrumental in driving beiging and conferring resistance to obesity in this system.

### AT-resident eosinophils are altered in the absence of KLF3

**Fig. 4:**
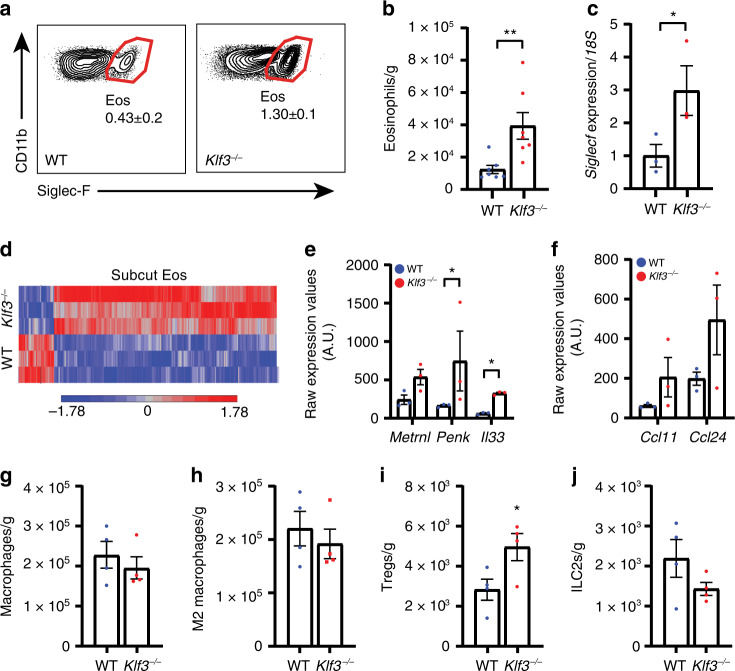
AT-resident eosinophils are altered in the absence of KLF3. **a** Flow cytometry was used to analyse the abundance of Siglec-F^+^ eosinophils in the SVF of WT and *Klf3*^*−/−*^ subcut AT. Representative plots of Siglec-F^+^ eosinophils as a percentage of live cells are shown accompanied by the means ± SEM of *n* = 3 mice per genotype. **b** The total number of eosinophils per gram of subcut AT was calculated by sorting the entire SVF from WT and *Klf3*^*−/−*^ mice (*n* = 7 mice). **c** mRNA levels of the murine eosinophil surface marker *Siglecf* were assessed by qPCR (*n* = 3 mice). Relative expression was normalised to levels of *18S* rRNA. **d** Eosinophils isolated from the subcut SVF of WT and *Klf3*^*−/−*^ mice by FACS were subjected to RNA extraction for microarray analysis, available at GEO (Accession No. GSE117445) (*n* = 3 mice). A heatmap was constructed showing significantly deregulated genes (*P* < 0.05, two-way ANOVA using false discovery rate, FDR, to correct for multiple comparisons). **e** Three genes with reported roles in beige fat activation and AT homeostasis were identified as significantly upregulated in *Klf3*^*−/−*^ AT eosinophils and their raw expression values were calculated by robust multi-array average (RMA) normalisation, with error bars representative of the means ± SEM of *n* = 3 mice per genotype. **f** Raw expression values for eotaxins *Ccl11* and *Ccl24* were also calculated in WT and *Klf3*^*−/−*^ subcut AT eosinophils (*n* = 3 mice). Abundance of **g** macrophages (live CD45^+^CD11b^+^CD64^+^ cells^)^ and **h** M2 macrophages (live CD45^+^CD11b^+^CD64^+^CD206^+^CD11c^−^ cells) in the SVF of WT and *Klf3*^*−/−*^ subcut AT was determined by flow cytometry and normalised to tissue weight (*n* = 4 mice). Abundance of **i** Tregs (live CD45^+^CD19^-^CD3^+^CD4^+^CD25^+^ cells) and **j** ILC2s (CD45^+^Lin^−^ CD127^+^CD25^+^ cells) were also determined by flow cytometry and normalized to tissue weight (*n* = 4 mice). For **b**, **c** and **e**–**j** error bars represent means ± SEM and one-sided non-parametric Mann–Whitney *U* tests were performed where *P < 0.05, **P < 0.01. Source data are provided as a Source data file. Eos, eosinophils; Tregs, regulatory T cells; ILC2s, group 2 innate lymphoid cells; A.U., arbitrary units. See also Supplementary Figs. 6–8.

Given that transplantation with KLF3-deficient haematopoietic cells promoted resistance to obesity, we sought to identify the important cell types in this process. *KLF3* is expressed in multiple tissues and cell lineages, including various haematopoietic cells^34,35^, and the FANTOM5 SSTAR database that measures transcript levels indicates it is more abundant in human eosinophils than any other cell type^37^, consistent with its observed presence in murine eosinophils^38^ (Supplementary Fig. 6a). Given their proposed role in adiposity and thermogenesis^3–5,39–41^, we sought to assess the contribution of eosinophils in driving the thermogenic phenotype of *Klf3*^*−/−*^ mice. We first examined the abundance of eosinophils in subcut AT of WT and *Klf3*^*−/−*^ mice. SVF cells were collected and analysed by flow cytometry (according to the eosinophil gating strategy in Supplementary Fig. 7a). AT-resident eosinophils were 3-fold more prevalent in *Klf3*^*−/−*^ subcut AT (Fig. 4a). We also sorted eosinophils from whole subcut AT to determine the number of cells per gram and confirmed that eosinophil abundance is greater in *Klf3*^*−/−*^ subcut AT (Fig. 4b). mRNA levels of the gene encoding the eosinophil surface marker Siglec-F (*Siglecf*) were also 3-fold higher in *Klf3*^*−/−*^ subcut AT (Fig. 4c). It is noteworthy that while eosinophils are more than 2-fold more abundant in *Klf3*^*−/−*^ gonadal AT (Supplementary Fig. 7b), no differences in the number of lung or spleen eosinophils were detected (Supplementary Fig. 7c, d). Eosinophils were also found to be over 2-fold more prevalent in the subcut AT of WT^*Klf3−/−*^ chimeric mice than in WT^WT^ counterparts on a Western diet (Supplementary Fig. 7e, f). We next compared the genome-wide expression profiles of eosinophils isolated from subcut SVF by FACS and observed broad deregulation of gene expression in the absence of KLF3 (Fig. 4d), resulting in changes to important cellular pathways including signalling, localisation, response to stimulus and immune system processes (Supplementary Fig. 6b). Further breakdown of the deregulated immune system processes in *Klf3*^*−/−*^ eosinophils revealed striking enrichment of various biological pathways such as leukocyte migration and myeloid cell homeostasis (Supplementary Fig. 6c). To explore the transcriptional network further we interrogated the transcriptional activator *Klf1*, which operates in a regulatory system with *Klf3* in erythroid cells^32,42^. *Klf1* was very lowly expressed in AT eosinophils and showed no difference between WT and *Klf3*^*−/−*^ (Supplementary Fig. 6d, e), with no expression detected in whole AT or SVF (Supplementary Fig. 6f), suggesting that KLF1 does not play an important opposing role to KLF3 in AT eosinophils. Inspection of the most significantly deregulated genes in KLF3-deficient adipose eosinophils revealed three genes encoding proteins with reported roles in beige AT activation—meteorin-like (*Metrnl*)^5^, met-enkephalin (*Penk*)^14,16,43^ and IL-33 (*Il33*)^14,15^ (Fig. 4e). We have previously reported that levels of galectin-3, a proposed eosinophil chemoattractant^44,45^, are elevated in *Klf3*^*−/−*^ AT^29^. Here we found that the eosinophil chemoattractants eotaxin-1 and -2 (*Ccl11* and *Ccl24*) were upregulated in KLF3-deficient AT eosinophils (Fig. 4f), providing a possible explanation for the increased eosinophil abundance in *Klf3*^*−/−*^ AT. Given that eosinophil abundance is elevated in *Klf3*^*−/−*^ AT, we sought to quantify other immune cells in the stromal vascular compartment. We did not observe changes to macrophage abundance (M1 or M2), dendritic cells, CD19^+^ B cells or CD3^+^ T cells (Fig. 4g, h and Supplementary Fig. 8a–e). T regulatory lymphocytes (Tregs) were more abundant in KLF3-deficient subcut AT (Fig. 4i and Supplementary Fig. 8a)—unsurprising given the elevated expression of IL-33^46–49^. While no significant differences to the overall lineage^−^ CD127^+^ CD25^+^ group 2 innate lymphoid cell (ILC2) population were observed, we did detect a significant deficit of ILC2s expressing the IL-33 receptor (ST2) in *Klf3*^*−/−*^ subcut AT (Fig. 4j and Supplementary Fig. 8a, f, g). To assess whether changes in the AT immune profile affected cytokine signalling, we measured expression of various inflammatory and anti-inflammatory genes in the SVF of WT and *Klf3*^*−/−*^ subcut AT. Expression of the classical inflammatory marker *Tnf* (encoding the pro-inflammatory molecule TNF-α) was reduced in the absence of KLF3, while the M2 macrophage marker *Arg1* was increased (Supplementary Fig. 7g). Unexpectedly, we detected increased expression of *Ifng* (encoding the inflammatory cytokine interferon-γ) in *Klf3*^*−/−*^, which has been shown to block themogenesis^50^. On the other hand, levels of *Il10* were reduced in the absence of KLF3, in line with recent findings that IL-10 blockade protects against diet-induced obesity and elicits browning of AT^51^. Together these findings demonstrate that resident eosinophils are more abundant in *Klf3*^*−/−*^ AT and that these cells exhibit widespread deregulated gene expression, including higher levels of genes implicated in beige AT activation. These changes are accompanied by modifications to the immune and cytokine landscape of subcut AT.

### KLF3 directly regulates AT eosinophil gene expression

**Fig. 5:**
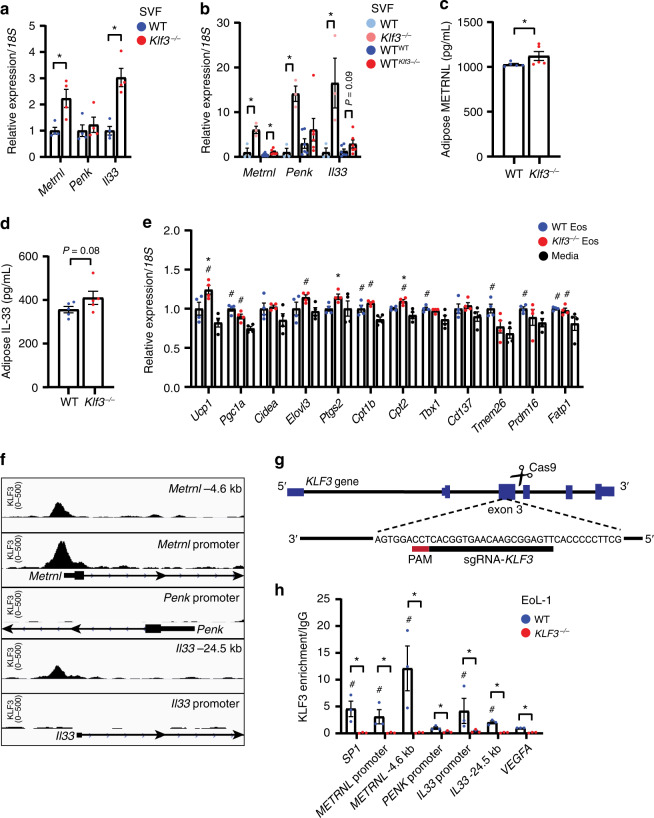
KLF3 directly regulates AT eosinophil gene expression. **a** *Metrnl*, *Penk* and *Il33* mRNA levels in subcut SVF from **a** WT and *Klf3*^*−/−*^ mice (*n* = 4 mice) and **b** WT, *Klf3*^*−/−*^ (*n* = 3), WT^WT^ and WT^*Klf3−/−*^ (*n* = 6) mice. Relative expression was normalised to *18S* rRNA and WT values set to 1 for each gene. Levels of secreted **c** meteorin-like and **d** IL-33 were measured in supernatants of WT and *Klf3*^*−/−*^ AT explants by ELISA (*n* = 5 mice). **e** Expression of thermogenic genes in differentiated preadipocytes co-cultured for 4 h with WT or *Klf3*^*−/−*^ eosinophils (or media alone) was measured by qPCR (*n* = 4 biological replicates). Relative expression was normalised to *18S* and WT eosinophil-treated values set to 1. **f** Tracks at the promoter and upstream regions of *Metrnl*, *Penk* and *Il33* were obtained from a KLF3-V5 ChIP-Seq in murine embryonic fibroblasts (GEO Accession No. GSE44748) to assess in vivo KLF3 binding. Scale is shown on the left (KLF3 0-500) and regions are denoted in the top right corner. **g** CRISPR/Cas9 strategy used to delete *KLF3* in EoL-1 cells. **h** ChIP-qPCR was performed in WT and *KLF3*^*−/−*^ human EoL-1 cells to assess direct binding of KLF3 to *Metrnl*, *Penk* and *Il33* (*n* = 3 independent experiments). KLF3 enrichment is normalised to input (non-immunoprecipitated DNA) and a goat IgG control, with human *SP1* and *VEGFA* included as positive and negative control loci respectively. *KLF3*^*−/−*^ EoL-1 cells were used to confirm the affinity of the anti-KLF3 antibody to KLF3 antigen, with no enrichment expected in these cells. For **a**–**d** one-sided non-parametric Mann–Whitney *U* tests were performed where *P < 0.05. For **e** one-sid**e**d non-parametric Mann–Whitney *U* tests were performed where *P < 0.05 between WT and *Klf3*^*−/−*^ eosinophil co-culture, and ^#^*P* < 0.05 between WT or *Klf3*^*−/−*^ eosinophil co-culture and media alone treatment. For **h** one-sided non-parametric Mann–Whitney *U* tests were performed where *P < 0.05 between WT and *KLF3*^*−/−*^ for each region and #*P* < 0.05 relative to the WT *VEGFA* negative control. For **a**–**e** and **g**, **h**, error bars represent means ± SEM. Source data are provided as a Source data file. Eos, eosinophils; kb, kilobase pairs; PAM, protospacer adjacent motif. See also Supplementary Fig. 9.

Our finding that AT eosinophils lacking KLF3 express higher levels of *Metrnl*, *Penk* and *Il33* led us to examine these genes more closely. Using qPCR analysis, we measured higher expression of *Metrnl* and *Il33* in subcut SVF from *Klf3*^*−/−*^ compared to WT mice (Fig. 5a). Likewise, we found that levels *Metrnl* and *Il33* were higher in WT^*Klf3−/−*^ subcut SVF than in WT^WT^ (Fig. 5b). Interestingly, *Penk* expression was only elevated in subcut SVF from *Klf3*^*−/−*^ mice fed a Western diet, with no differences seen in whole subcut AT or SVF from transplanted mice or from mice fed a chow diet (Fig. 5a, b and Supplementary Fig. 8a, b). To assess levels of secreted meteorin-like and IL-33 in AT, we quantified their concentration in supernatant from AT explants cultured for 2 h, using ELISA. We found that both meteorin-like and IL-33 were more highly concentrated in *Klf3*^*−/−*^ AT supernatant, suggesting greater secretion of these proteins (Fig. 5c, d). However, when we measured meteorin-like and IL-33 in peripheral blood plasma we found no difference (Supplementary Fig. 8c, d), which may suggest that these proteins are elevated locally and have a paracrine effect confined to the AT microenvironment. To determine whether AT eosinophils are able to directly drive beiging of adipocytes, we performed co-culture experiments. FACS-sorted eosinophils from WT and *Klf3*^*−/−*^ subcut SVF were co-cultured with mature primary adipocytes for 4 h, and the expression of thermogenic genes measured by qPCR (Fig. 5e). We observed modest but significant increases in the expression of several thermogenic genes in adipocytes co-cultured with WT eosinophils compared to media alone. Additionally, several genes, including *Ucp1*, *Ptgs2* and *Cpt2*, were more highly expressed in adipocytes co-cultured with *Klf3*^*−/−*^ AT eosinophils than WT eosinophils. Together these results suggest that AT eosinophils alone are able to drive beiging of adipocytes, at least in vitro, and that KLF3 may directly regulate gene expression in eosinophils. Given that in the absence of the transcriptional repressor KLF3, *Metrnl* and *Il33* are upregulated and secreted in higher abundance within AT, we sought to determine whether KLF3 directly binds and regulates these genes. Inspection of ChIP-Seq data from mouse embryonic fibroblasts (Accession No. GSE44748)^52^ showed distinct KLF3 binding at the *Metrnl* promoter region, but little enrichment at *Penk* or *Il33* (Fig. 5f). To assess whether KLF3 binds these genes in a relevant cellular setting we performed ChIP in WT and *KLF3*^*−/−*^ EoL-1 cells, a human eosinophilic cell line that highly expresses *KLF3* (Supplementary Fig. 8e). *KLF3*^*−/−*^ cells were generated by CRISPR/Cas9 non-homologous end joining following the introduction of a sgRNA targeting exon 3 of *KLF3* (Fig. 5g). After undertaking immunoprecipitations with an anti-KLF3 antibody or normal goat IgG, we performed qPCR using primers designed to amplify the promoter regions of *METRNL*, *PENK* and *IL33*, and to positive control regions based on known ChIP-Seq peaks (*SP1*, *METRNL* −4.6 kb*, IL33* −24.5 kb) and a negative control locus (*VEGFA*). We found KLF3 binding was evident at the *METRNL* and *IL33* promoters compared to the negative control region *VEGFA*, and that signals in *KLF3*^*−/−*^ samples were negligible, confirming the specificity of the anti-KLF3 immunoprecipitation (Fig. 5h). This indicates that KLF3 directly binds and regulates the expression of the genes encoding important secreted molecules in adipose-resident eosinophils.

## Discussion

Type 2 immune cells are now recognised as key players in AT homeostasis and beiging. Much attention has been given to the contribution of macrophages^4,8,53,54^, however recent findings suggest that AT macrophages may not perform their previously-defined direct contribution to adaptive thermogenesis via catecholamine production^9–11^. This realisation provides us with an opportunity to re-evaluate the current model of type 2 immunity in adiposity and beiging by defining previously unknown or under-appreciated immune cells and the mechanisms underpinning their contribution to AT homeostasis. A recent study found that AT immune cells secrete acetylcholine that elicits beiging, highlighting the importance of haematopoietic cells in AT homeostasis^55^. Interestingly though, acetylcholine-producing cells were predominantly B cells, T cells and macrophages, leaving the contribution of other lineages, such as eosinophils, unresolved. In this study, using a combination of in vitro approaches, transgenic mice and BM transplantation models, we have shown that the transcriptional repressor KLF3 directly regulates genes encoding secreted factors in AT eosinophils. Our findings from co-culture experiments suggest that eosinophils may be able to directly drive activation of beiging in adipocytes through paracrine signalling. This highlights the importance of eosinophils in our model of type 2 immunity in AT beiging and suggests that eosinophils may be able to activate beige fat in an organismal setting, providing protection from obesity. Future work will seek to further characterise the in vivo contribution of eosinophils to adipose homeostasis and thermogenesis, and to understand the relevance of these cells in human setting.

BM transplantation studies have been utilised to explore the contribution of haematopoietic cells in adiposity and beiging, including the metabolic response to caloric restriction^56^ and hypermetabolism following burn injury^57^. We cannot exclude the influence of other haematopoietic cells to the reduced weight gain and improved metabolic parameters in WT^*Klf3−/−*^ mice, and while this work explores the contribution of eosinophils, considerable effort in the field is currently being directed towards understanding the diverse roles of various immune cell populations in beiging. Although eosinophil infiltration has been implicated in beiging^3,40^, it is also apparent that artificially increasing their abundance by administration of IL-5 does not appear to significantly improve major metabolic parameters in diet-induced obese mice^7^. The authors proposed that eosinophil activity may be more important than total numbers in AT. Significantly, as well as observing increased eosinophil abundance in *Klf3*^*−/−*^ AT, we found considerable deregulation of biological pathways in these cells and upregulation of genes encoding key secreted factors that activate beige AT. Meteorin-like is secreted by exercised muscle and induced in AT following cold exposure^5^, however, a comprehensive understanding of the AT-specific source has remained elusive. Recent studies have reported adipose stromal cells as the primary source of IL-33, with only a small contribution potentially originating from hematopoietic cells^48,49^. Here we provide evidence that eosinophils may contribute a portion of local adipose IL-33, as well as meteorin-like. While the absence of *Penk* deregulation in chow-fed *Klf3*^*−/−*^ mice and lack of evidence for direct binding by chromatin immunoprecipitation led us to emphasize other putative target genes, we cannot rule out a direct role for KLF3 in the regulation of *Penk*, or a role for Met-Enk in contributing to the phenotype observed—potentially in other cell types.

**Fig. 6:**
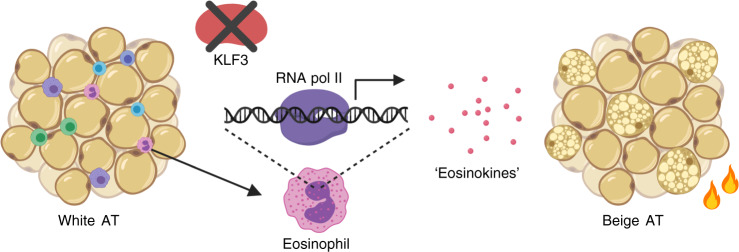
KLF3 regulation of eosinophil gene expression and function in adipose tissue. Proposed model by which KLF3 may regulate the expression of eosinophil-derived factors in the conversion from white to beige AT.

Overall, our findings demonstrate a mechanism by which type 2 immune cells may influence beiging and adiposity—via KLF3-driven regulation of important eosinophil-derived factors that we term ‘eosinokines’^40^, as shown in our proposed model (Fig. 6). Our results not only suggest that eosinophils may play an under-appreciated role as signalling cells in metabolism, but also reveal that KLF3 is a key regulator of secreted molecules in eosinophils. Multiple KLF family members have been implicated in the control of metabolism^58–62^ but few clear unifying models of action have emerged. KLF3, for instance, does not appear to regulate meteorin-like or IL-33 in other cell types, and indeed its role in eosinophils has previously been unrecognised. Targeting molecules secreted by immune cells and their receptors may have the potential to therapeutically drive energy expenditure via AT beiging to combat obesity. Our findings advance the search for secreted factors produced by eosinophils and other resident haematopoietic cells, and support the hypothesis that eosinophils and their secreted ‘eosinokines’ are relevant to the type 2 immune network in AT.

## Methods

### Animal husbandry

All animal work was carried out in accordance with approval from the UNSW Animal Care and Ethics Committee (Approval Nos. 12/150A, 16/5B and 16/141B), the Murdoch Children’s Research Institute Animal Ethics Committee (Approval No. A760) and the University of Sydney Animal Care and Ethics Committee (Approval No. L02/7-2009/3/5054). Animals were housed in a specific pathogen-free environment at a constant ambient temperature of 22 °C and 50% humidity on a 12 h light–dark cycle, with *ad libitum* access to standard chow food and water, unless otherwise specified. Generation of global *Klf3*^*−/−*^ mice on an FVB/NJ background has been previously reported^13^. Age-matched WT and *Klf3*^*−/−*^ male littermates derived from *Klf3*^*+/*−^ × *Klf3*^*+/*−^ crosses were used for all animal studies. Male mice were housed in cages containing bedding, nesting material and enrichment with up to five individual animals, except for cold and thermoneutral experiments which were undertaken in empty cages containing singly housed mice.

### Acute thermoneutral and cold temperature exposure

For acute temperature exposure experiments, WT and *Klf3*^*−/−*^ mice aged between 12 and 14 weeks were assessed at 30 °C or 4 °C for 5 h. Mice housed at thermoneutrality (30 °C) were acclimatised at this temperature for 20 h prior to commencement of experiments. Food, water and bedding were removed from cold-exposed mice during the 5 h period. Body temperatures were obtained by rectal probe (BAT-12 microprobe thermometer) over a 5 h period between the times of 0800 h and 1400 h. Temperatures were measured at 0, 30, 60, 90, 120, 180, 240 and 300 min. Body weights were recorded before and after the 5 h temperature measurement period. At the conclusion of the 5 h assessment, all mice were euthanised and tissues and blood were collected for further analysis.

### Bone marrow transplantation studies

WT mice aged 7 weeks old were irradiated using an X-RAD 320 with two doses of 500 cGy, 6 h apart. The following day, 7-week old WT and *Klf3*^*−/−*^ donor mice were euthanised and femora and tibiae harvested. BM cells were flushed with sterile RPMI medium and red blood cells lysed using distilled and deionised water. Cells were resuspended in phosphate-buffered saline (PBS) and adjusted to 5 × 10^7^/mL. Recipient mice were warmed using infrared heat lamps then 1 × 10^6^ cells were injected into the tail vein. Transplantation recipients were administered with antibiotics containing 200 mg/L sulfamethoxazole and 40 mg/L trimethoprim via drinking water for 2 weeks. Following this, recipient mice and a control cohort of WT and *Klf3*^*−/−*^ mice were transferred to a high-fat, high-sugar Western diet for 11 weeks (Supplementary Table 1). Mice were weighed and assessed for body mass composition using an EchoMRI before and throughout the study, and at the conclusion tissues and blood were collected for further analyses. Genotyping of tail biopsies and BM was performed to confirm reconstitution of WT or *Klf3*^*−/−*^ cells in the BM of chimeric mice. Genotyping primers can be found in Supplementary Table 2, and gel images were analysed using Bio-Rad Image Lab software v6.0.1.

### Blood and plasma analysis

Peripheral blood was collected by cardiac bleed using 50 U/mL heparin sulphate as an anti-coagulant, and stored in K2EDTA collection tubes. Whole blood was sent for biochemical analysis of triglycerides and total cholesterol at the University of Sydney Veterinary Pathology Diagnostic Services. Plasma was isolated by centrifugation (2000 × *g* for 15 min) of whole blood and stored for further analysis.

### Mouse tissue processing

AT was harvested from the posterior subcutaneous white AT depot (composed of dorsolumbar, inguinal and gluteal portions), gonadal white AT depot and the interscapular brown AT depot. To obtain the AT stromal vascular fraction (SVF), depots were minced then digested using 1 mg/mL type II collagenase and cells were passed through a 40 μm sieve to remove undigested particles. The same digestion procedure was applied for lung tissue with the addition of 1 μg/mL DNaseI. Spleens were homogenised in cold PBS. Centrifugation at 500 × *g* for 10 min was then performed to separate the SVF pellet from floating adipocytes, for downstream usage. AT explants were performed^25^, with supernatants harvested after 2 h of culture before being frozen and stored for further analysis.

### Adipose H&E staining

Sections of subcut AT were mounted and stained with haematoxylin and eosin according to routine protocols. H&E sections were imaged on an Aperio XT Slide Scanner and processing was undertaken using Aperio ImageScope software.

### Liver biochemical assays

Liver was snap-frozen in liquid nitrogen and stored at −80 °C until lipid extraction. Lipids were extracted from powdered tissue using a modified Folch method^63^. The lipid extract was dried under a steady-stream of nitrogen. Extracts were resuspended in 0.4 mL 95% ethanol and heated to 37 °C prior to lipid assays. Colorimetric assays were used to measure triglycerides (Point Scientific) and cholesterol (Thermo Fisher). All assays were conducted according to the manufacturer’s protocols and results were normalised to liver weight.

### Cell culture

All cell lines were incubated in a 37 °C 5% CO_2_ water-jacketed incubator. COS-7 cells were a gift from Stuart Orkin (Harvard), and were cultured in DMEM supplemented with 10% foetal bovine serum (FBS) and 1% penicillin–streptomycin–glutamine (PSG). During passaging, adherent cells were lifted after a 5 min incubation at 37 °C with 2 mM PBS-EDTA. EoL-1 cells were grown in RPMI 1640 supplemented with 10% FBS and 1% PSG. The EoL-1 cell line was supplied by the European Collection of Cell Cultures (ECACC; Salisbury, United Kingdom) as catalogue number 94042252, and purchased from Sigma Aldrich. The cell line has been reported before^64^.

### Genome editing

For CRISPR-Cas9 genome editing, a plasmid encoding both the Cas9 protein and the sgRNA was used to delete *KLF3* in EoL-1 cells via double-strand breakage and non-homologous end joining. pSpCas9(BB)-2A-GFP (PX458) was a gift from Feng Zhang (Addgene plasmid 48138)^65^. We designed sgRNA sequences using the Optimized CRISPR-Cas9 design v1 online tool provided by the Zhang laboratory from the Massachusetts Institute of Technology. EoL-1 cells were transfected by nucleofection using a Neon Transfection System (Life Technologies). Cells (5 × 10^5^) were resuspended in nucleofection buffer R (Neon Transfection Kit) and given one pulse of 1350 V for 30 ms. Cells were then cultured for 72 h in RPMI 1640 with 10% FBS but lacking antibiotics. Transfected cells were enriched by FACS, and clonal populations were established by sorting single cells into 96-well culture plates. To screen clones for the desired *KLF3* disruption, PCR was carried out on genomic DNA using Q5 polymerase (New England BioLabs), before confirmation via Sanger sequencing of PCR products. sgRNA, PCR and sequencing oligonucleotides can be found in Supplementary Table 2.

### Flow cytometry

Flow cytometry was performed using a BD LSRFortessa and BD LSRFortessa X-20. Sorting by FACS was performed using a BD Influx and BD FACS Aria II. All cells were pre-blocked with anti-CD16/32 Fc block to reduce non-specific binding, and UltraComp eBeads (Invitrogen) were used for single-stained compensation controls. For identifying eosinophils, cells were stained with a combination of the following antibodies: CD45-biotin (BD Pharmingen), Streptavidin-BV711 (BD Horizon), CD11b-FITC (BD Pharmingen), F4/80-PE/Cy7 (Biolegend) and SiglecF-BV421 (BD Horizon). Live cells were identified by the addition of TO-PRO-3 viability dye. Eosinophils were defined as live CD45^+^ CD11b^+^ F4/80^+^ Siglec-F^+^ SSC^hi^ cells. For delayed flow cytometric analysis, fully stained cells were fixed with 1% paraformaldehyde. In these instances, ZombieNIR (Biolegend), a fixable viability dye, was used for identifying live cells, and F4/80 was conjugated to PE/Cy5 (eBioscience). To analyse other AT immune cell populations, various fluorescently-conjugated antibodies were used and detailed antibody information can be found in Supplementary Table 3, with gating strategies for immune cell populations available in the legends for Fig. 4 and Supplementary Fig. 8. Flow cytometry analysis was performed using FlowJo software v10.

### Co-culture experiments

Culture and differentiation of primary mouse adipocytes was performed^66^. Briefly, isolated SVF cells from the inguinal subcutaneous fat of WT mice were differentiated in 12-well plates for two days using an induction medium of DMEM-F12 GlutaMAX with 10% FBS and 1X PSG, 1 μg/mL insulin, 0.5 mM IBMX, 0.25 μM dexamethasone, 1 μM rosiglitazone and 1 nM triiodothyronine (T3). Two days after induction, cells were cultured in maintenance medium (DMEM-F12 GlutaMAX containing 10% FBS, 1X PSG, 1 μM rosiglitazone, 1 nM T3 and 1 μg/mL insulin) which was refreshed every two days. Co-culture experiments were performed in a bicompartmental system using 0.4 μm pore polycarbonate transwell inserts (Corning). A standardised number of adipose eosinophils were sorted from WT and *Klf3*^*−/−*^ subcutaneous SVF using a BD FACS Aria III then resuspended in RPMI containing 10% FBS, 1X PSG and 10 ng/mL IL-5. Eosinophils (or media alone) were then added to the upper compartment of the transwell and incubated at 37 °C in 5% CO_2_ for 4 h. Adipocytes from the lower compartment were then collected for gene expression analysis by qPCR.

### Gene expression analysis

To assess mRNA expression, total RNA was isolated from cells and tissues then subjected to cDNA synthesis^29^. Quantitative real-time PCR (qPCR) reactions were set up with Power SYBR Green PCR Master Mix and were run with default cycle parameters on the Applied Biosystems 7500 Fast Real-Time PCR System (for 96-well plate format) or the Applied Biosystems ViiA7 Real-Time PCR System (for 384-well plate format). Applied Biosystems 7500 software v2.3 and Applied Biosystems QuantStudio Real-Time PCR software v1.3 were used for qPCR data analysis. Gene expression was quantified using the 2^−ΔΔCT^ method and relative mRNA expression was normalised to *18S* rRNA levels which have been shown to display consistent expression across the cells and conditions studied. All qPCR primers were designed using primer3 (http://primer3.ut.ee/) and can be found in Supplementary Table 2. For microarrays on eosinophils sorted from subcut SVF by FACS, RNA was isolated using the RNeasy Micro Kit (Qiagen) then subjected to quality control using an Agilent 2100 Bioanalyzer, following preparation with an Agilent RNA 6000 Pico Kit. An Affymetrix 3’ IVT Pico Kit was used prior to microarrays which were performed on an Affymetrix GeneChip HT MG-430PM Array Plate. Partek Genomics Suite v7 software was used for data analysis, and microarray datasets are available from GEO (Accession No. GSE117445).

### Protein extraction and quantification

Whole-cell protein extracts (WCE) were prepared by homogenising mouse tissues with a glass dounce, in the presence of radioimmunoprecipitation (RIPA) buffer (50 mM HEPES, pH 7.5; 500 mM LiCl; 1 mM EDTA; 1% NP-40; 0.7% sodium deoxycholate) containing protease inhibitors (cOmplete, Mini, EDTA-free Protease Inhibitor Cocktail; one tablet dissolved in 10 mL RIPA buffer). Homogenates were rotated at 4 °C for 1 h then centrifuged at 21,000 × *g* for 20 min at 4 °C to obtain the WCE. Protein extracts were subjected to a bicinchoninic acid (BCA) assay using a BCA Protein Assay Kit (Pierce) to determine concentration according to the manufacturer’s protocol. For detection of UCP1, 25 μg of WCE was loaded onto Novex NuPAGE 10% Bis-Tris gels, following denaturation and boiling (15 μg for brown AT WCE). After blocking, nitrocellulose membranes were incubated overnight with anti-UCP1 antibody (Abcam). Membranes were then probed with HRP-linked anti-rabbit antibody (GE Healthcare) prior to exposure using the GE ImageQuant LAS 500 in the presence of Immobilon Western Chemiluminescent HRP Substrate (Millipore). To detect expression of VDAC (voltage-dependent anion channel), 20 μg of WCE was used and incubation took place overnight with anti-VDAC antibody (Cell Signaling) before probing with HRP-linked anti-rabbit secondary antibody. To detect mitochondrial electron transport chain complexes, several deviations from the above protocol were performed. Briefly, WCE were boiled for 5 min at 50 °C before SDS-polyacrylamide gel electrophoresis (PAGE) and transferral to a 0.45 μm polyvinyl difluoride (PVDF) membrane. Blocking took place overnight at 4 °C using 5% (w/v) skim milk powder in PBS (pH 7.4) with gentle agitation, followed by washing in PBST (PBS with 0.1% Tween-20). The membrane was incubated with the primary Total OXPHOS Rodent WB Antibody Cocktail (Abcam) in 1% skim (w/v)/PBS. This optimised primary antibody mix contains five mouse antibodies, one each against the mitochondrial oxidative phosphorylation (oxphos) complexes (Complex I: NADH dehydrogenase; Complex II: succinate dehydrogenase; Complex III: CoQH_2_-cytochrome *c* reductase; Complex IV: cytochrome *c* oxidase; Complex V: ATP synthase). The secondary HRP-linked anti-mouse antibody labelling was performed in 1% skim milk (w/v)/PBS before imaging. All membranes were stripped with 0.5 M NaOH then re-blocked with skim milk before probing with an anti-β-actin antibody (Sigma). Following this, HRP-linked anti-mouse antibody (GE Healthcare) was incubated on membranes prior to imaging. For measurement of meteorin-like and IL-33 levels in plasma and AT explant supernatant, mouse meteorin-like/METRNL DuoSet and mouse IL-33 DuoSet ELISA kits were utilised according to manufacturer’s instructions (R&D Systems). Detailed antibody information can be found in Supplementary Table 3.

### Chromatin immunoprecipitation

ChIP experiments were performed^67^. Briefly, ~7 × 10^7^ cells were used for each immunoprecipitation (IP). Cells were crosslinked with 1% formaldehyde for 10 min at room temperature, and the reaction was quenched with glycine at a final concentration of 125 mM. Crosslinked cells were then lysed and sonicated to obtain ~200–400 bp fragments of chromatin. Cross-linked DNA was pulled down at 4 °C overnight using 15 μg anti-KLF3 antibody (Thermo Fisher) or 15 μg control normal goat IgG (Santa Cruz Biotechnology) per IP. qPCR was performed on ChIP material using an Applied Biosystems ViiA7 Real-Time PCR System. For analysis, IPs were first normalised to the relative amount of input DNA, then to IgG controls. All qPCR primers for ChIP were designed using primer3 (http://primer3.ut.ee/) and can be found in Supplementary Table 2. A ChIP-Seq data set for V5-tagged KLF3 produced from murine embryonic fibroblasts was obtained from GEO (Accession No. GSE44748)^52^ and used to assess genome-wide KLF3 binding using Integrative Genomics Viewer (Broad Institute)^68,69^. Detailed antibody information can be found in Supplementary Table 3.

### Software

GraphPad Prism v8 was used for generation of graphs and statistical analysis. Adobe Photoshop and Illustrator CS5.1 were used for compiling and creating figures. FlowJo v10 was used for flow cytometry and cell sorting analysis. Applied Biosystems 7500 v2.3 and QuantStudio Real-Time PCR v1.3 were used for running and analysing qPCR experiments. Oligonucleotides were designed using the online primer3 tool v4.1.0, and the Optimized CRISPR design v1 online tool was used for designing sgRNAs. Integrative Genomics Viewer v2.4.9 (Broad Institute) was used for viewing ChIP-Seq data. Bio-Rad Image Lab v6.0.1 software was used for capturing and analysing agarose gels. Aperio ImageScope software was used for analysing H&E sections. Licensed BioRender.com online software was used for generating cartoons and figures.

### Statistical analysis

The mean is shown for data in each figure with individual data points shown, and SEM is shown as error bars. For flow cytometry plots, the median representative plot for each genotype/condition is shown, accompanied by the means ± SEM. D’Agostino-Pearson normality tests were performed to determine whether data followed a Gaussian distribution. Given the sample sizes, non-normality was determined, and thus non-parametric one-tailed Mann–Whitney *U* tests were used to test specific, directional hypotheses. *P < 0.05, **P < 0.01, ***P < 0.001 (or ^#^*P* < 0.05 where stated). Two-way ANOVA followed by post-hoc Tukey testing was carried out to assess significance between genotype, body temperature and time in acute cold and thermoneutral experiments. Two-way ANOVA followed by False Discovery Rate to test for multiple comparisons was used for significant differentially-expressed genes in microarray studies. Fisher’s Exact test was used to define significantly enriched GO terms in microarray Forest Plots. All statistical analyses were performed in GraphPad Prism v8.

### Reporting summary

Further information on research design is available in the Nature Research Reporting Summary linked to this article.

## Supplementary information

Supplementary Information

Reporting Summary

## Data Availability

Data supporting the findings of this study can be found within the paper and its Supplementary Information files, and a Source data file has been provided for data underlying figures. Further information is available upon reasonable request. Full scans of western blots are available in Supplementary Fig. 10. Datasets are publically available from the NCBI Gene Expression Omnibus (GEO) using Accession Nos. GSE117445 and GSE44748, FANTOM5 SSTAR https://fantom.gsc.riken.jp/5/sstar/Main_Page) and Haemosphere (haemosphere.org/). A reporting summary has also been provided as part of the Supplementary Information files. Source data are provided with this paper.

## Source data

Source Data
