# Supplementary material for: Eosinophil function in adipose tissue is regulated by Krüppel-like factor 3 (KLF3)
Source: Nat Commun. 2020 Jun 10;11:2922. doi: 10.1038/s41467-020-16758-9 (PMC7286919; doi:10.1038/s41467-020-16758-9)
Supplement: Supplementary file 1 — Supplementary Information [file 41467_2020_16758_MOESM1_ESM.pdf]

Supplementary Information

Eosinophil function in adipose tissue is regulated by Krüppel-like factor 3 (KLF3)

Knights et al.

Supplementary Fig. 1. Related to Fig. 1. Reduced adiposity and enhanced beiging in *Klf3*$^{-/-}$ mice.

Supplementary Fig. 2. Related to Fig. 1. Reduced adiposity and enhanced beiging in *Klf3*$^{-/-}$ mice.

Supplementary Fig. 3. Related to Fig. 2. KLF3 deficiency enhances the thermogenic response.

Supplementary Fig. 4. Related to Fig. 3. Increased beiging in WT mice transplanted with *Klf3*$^{-/-}$ bone marrow.

Supplementary Fig. 5. Related to Fig. 3. Increased beiging in WT mice transplanted with *Klf3*$^{-/-}$ bone marrow and evidence of additional metabolic effects in this mouse model.

Supplementary Fig. 6. Related to Fig. 4. AT-resident eosinophils are altered in the absence of KLF3.

Supplementary Fig. 7. Related to Fig. 4. AT-resident eosinophils are altered in the absence of KLF3.

Supplementary Fig. 8. Related to Fig. 4. AT-resident eosinophils are altered in the absence of KLF3.

Supplementary Fig. 9. Related to Fig. 5. KLF3 regulates AT eosinophil gene expression.

Supplementary Fig. 10. Western blot full scans.

Supplementary Table 1. Mouse diets.

Supplementary Table 2. Mouse and human oligonucleotides.

Supplementary Table 3. Antibodies

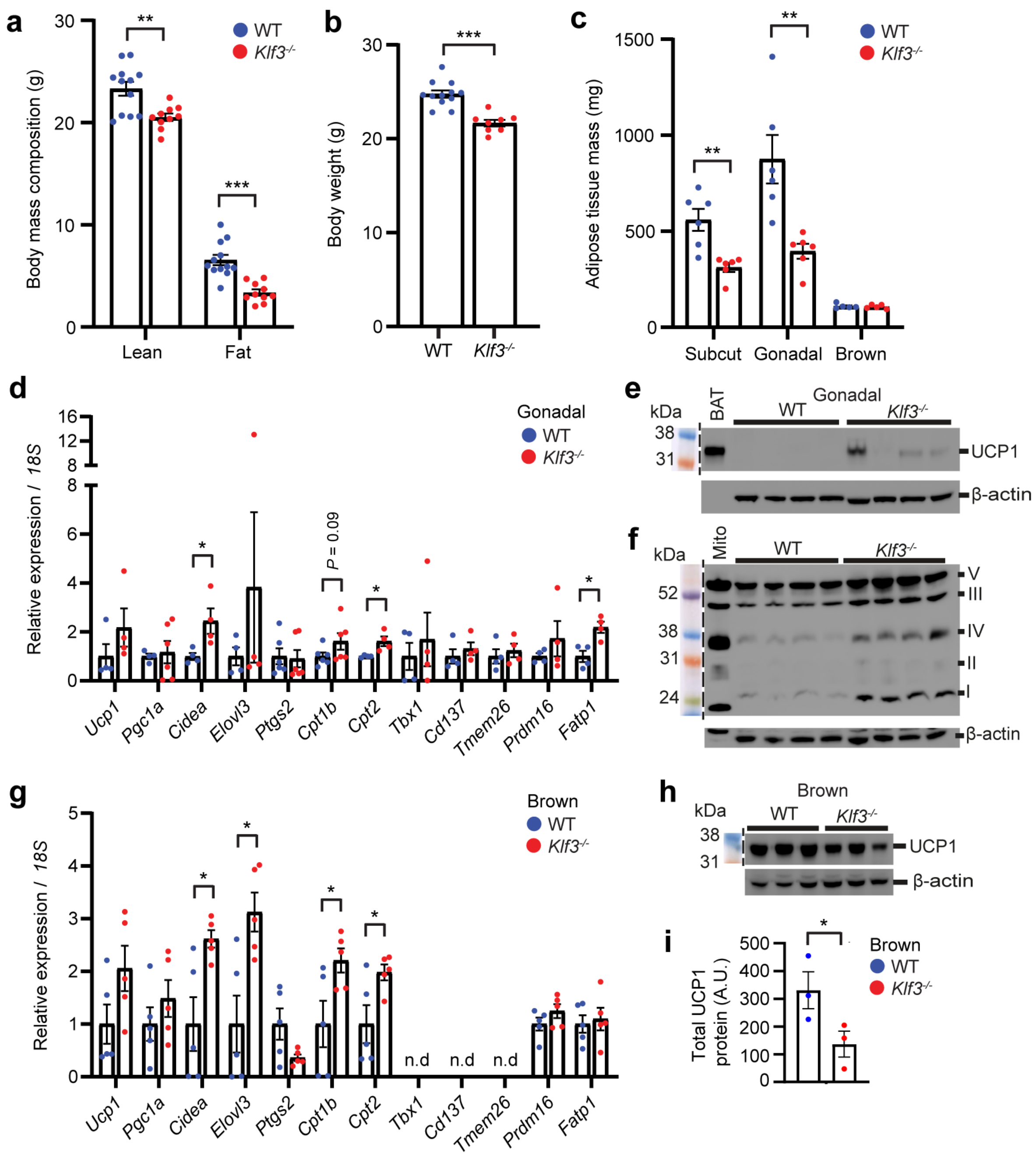

Supplementary Fig. 1. Related to Fig. 1. Reduced adiposity and enhanced beiging in *Klf3*$^{-/-}$ mice. a, lean and fat body mass composition (g) of WT (n=12) and *Klf3*$^{-/-}$ (n=10) mice were assessed by EchoMRI. **b**, body weights (g) of WT (n=12) and *Klf3*$^{-/-}$ (n=8) mice. **c**, weights (mg) of WT and *Klf3*$^{-/-}$ AT depots (n=6 mice for subcut and gonadal AT; n=5 mice for brown AT). **d**, mRNA levels of thermogenic genes were assessed by qPCR in WT and *Klf3*$^{-/-}$ gonadal AT (n=4-6 mice). Expression was normalised to *18S* rRNA levels and the WT value for each gene was set to 1. **e**, UCP1 protein was measured in WT and *Klf3*$^{-/-}$ gonadal AT by Western blotting (n=4 mice). 25 µg of WCE was loaded and nitrocellulose membranes were probed with anti-UCP1 overnight. Diluted brown AT (BAT) extract included as a positive control. **f**, expression of mitochondrial oxphos proteins in WT and *Klf3*$^{-/-}$ WCE was assessed by Western blotting (n=4 mice). 25 µg of extract was loaded alongside rat heart mitochondria extract (Mito; positive control), and PVDF membranes blocked overnight before being probed with the Total OXPHOS Rodent Antibody Cocktail for 2 h. Mitochondrial complexes are labelled I-V. **g**, mRNA levels of thermogenic genes were assessed by qPCR in WT and *Klf3*$^{-/-}$ brown AT (n=5 mice). Expression was normalised to *18S* rRNA levels and the WT value for each gene was set to 1. **h**, UCP1 protein was measured in WT and *Klf3*$^{-/-}$ brown AT by Western blotting (n=3 mice). 15 µg of WCE was loaded and nitrocellulose membranes were probed with anti-UCP1 overnight. For **e-f**, and **h**, β-actin was used as a loading control. **i**, total UCP1 protein content in WT and *Klf3*$^{-/-}$ brown AT depots normalized to tissue weight (n=3 mice). For **a-d**, **g** and **i**, error bars represent means ± SEM and one-sided non-parametric Mann-Whitney U tests were performed where *$P<0.05$, **$P<0.01$, ***$P<0.001$. Source data are provided as a Source Data file. n.d., not detected.

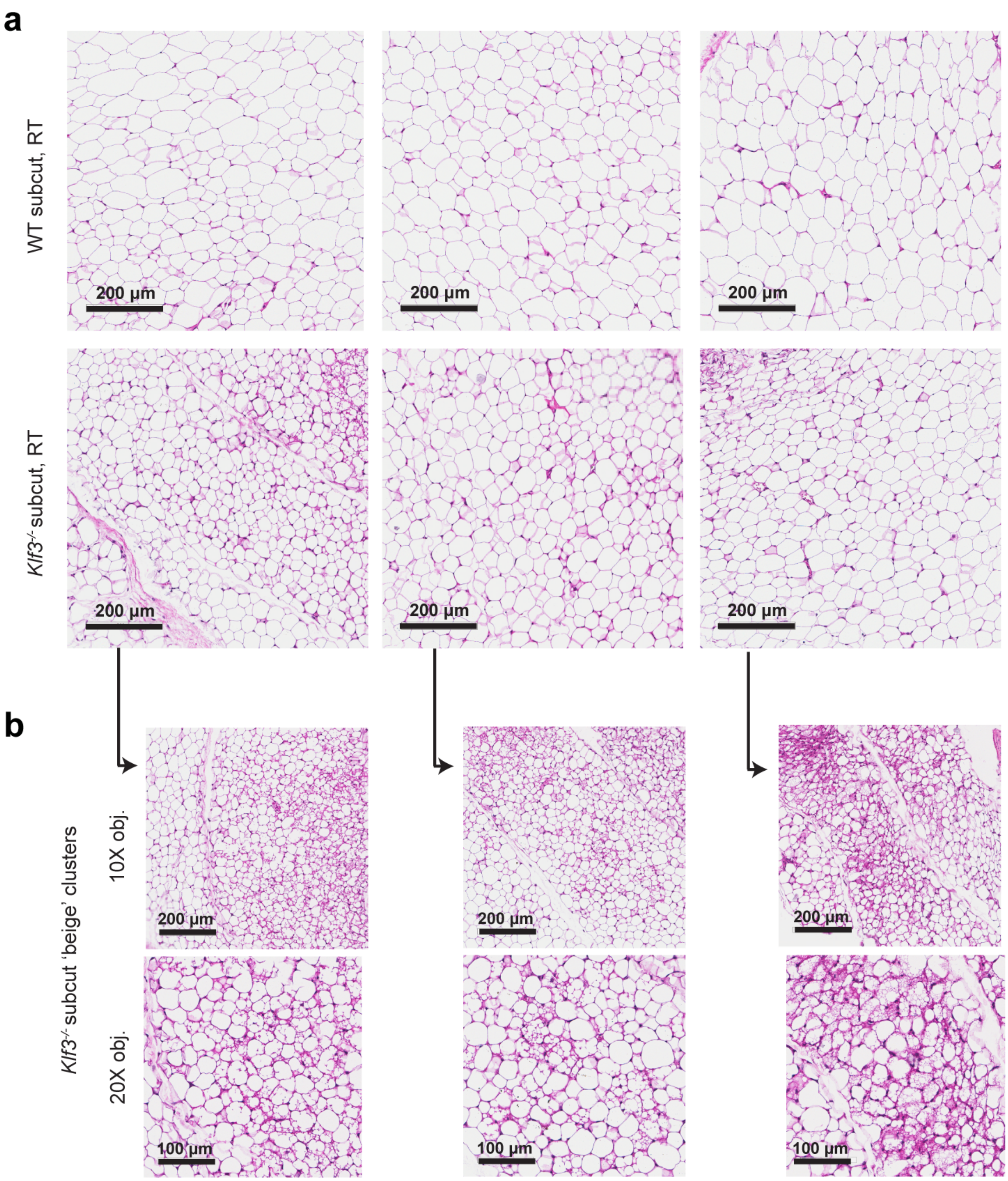

Supplementary Fig. 2. Related to Fig. 1. Reduced adiposity and enhanced beiging in *Klf3*$^{-/-}$ mice. a, H&E staining of subcut AT from WT and *Klf3*$^{-/-}$ mice housed at room temperature (RT) showing reduced adipocyte size, as has been previously reported in this mouse model (n=3 mice). 200 µm scale bar is shown, imaged at 10X magnification. **b**, clusters of enhanced 'beiging' in *Klf3*$^{-/-}$ subcut AT were observed (that were not evident in WT) at 10X and 20X magnification, showing multilocular lipid droplets. 200 µm and 100 µm scale bars are shown.

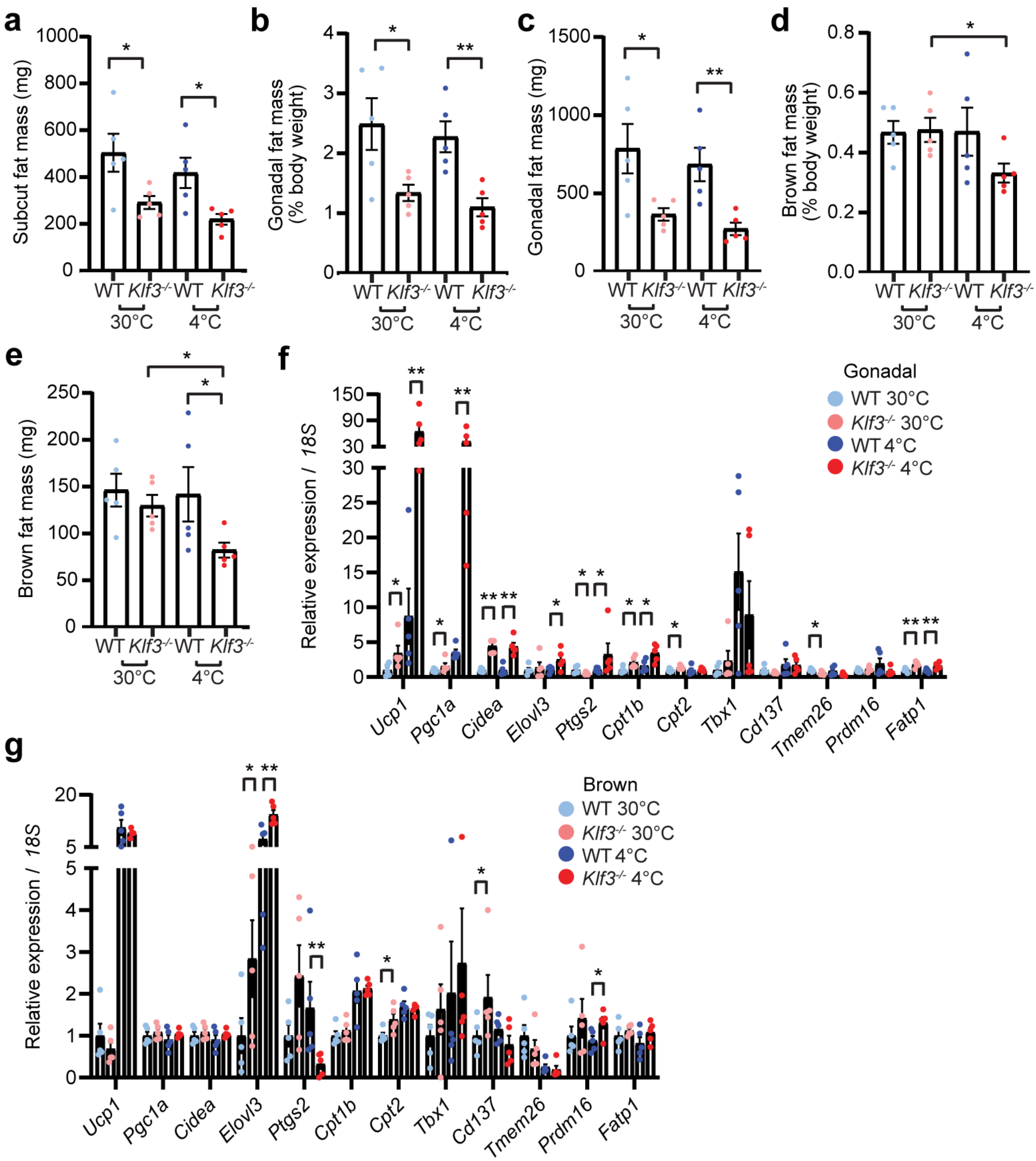

Supplementary Fig. 3. Related to Fig. 2. KLF3 deficiency enhances the thermogenic response. a, subcut (mg), **b**, gonadal (% body weight), **c**, gonadal (mg), **d**, brown (% body weight) and **e**, brown (mg) AT from WT and *Klf3*$^{-/-}$ mice housed at 30°C or 4°C were weighed (n=5 mice). mRNA levels of thermogenic genes were assessed by qPCR in WT and *Klf3*$^{-/-}$ **f**, gonadal and **g**, brown AT from mice housed at 30°C or 4°C (n=5 mice). Relative expression was normalised to *18S* rRNA levels and the WT 30°C value for each gene was set to 1. For **a-g**, error bars are representative of the means ± SEM and one-sided non-parametric Mann-Whitney U tests were performed to assess significance where **P*<0.05, ***P*<0.01. Source Data are provided as a Source Data file.

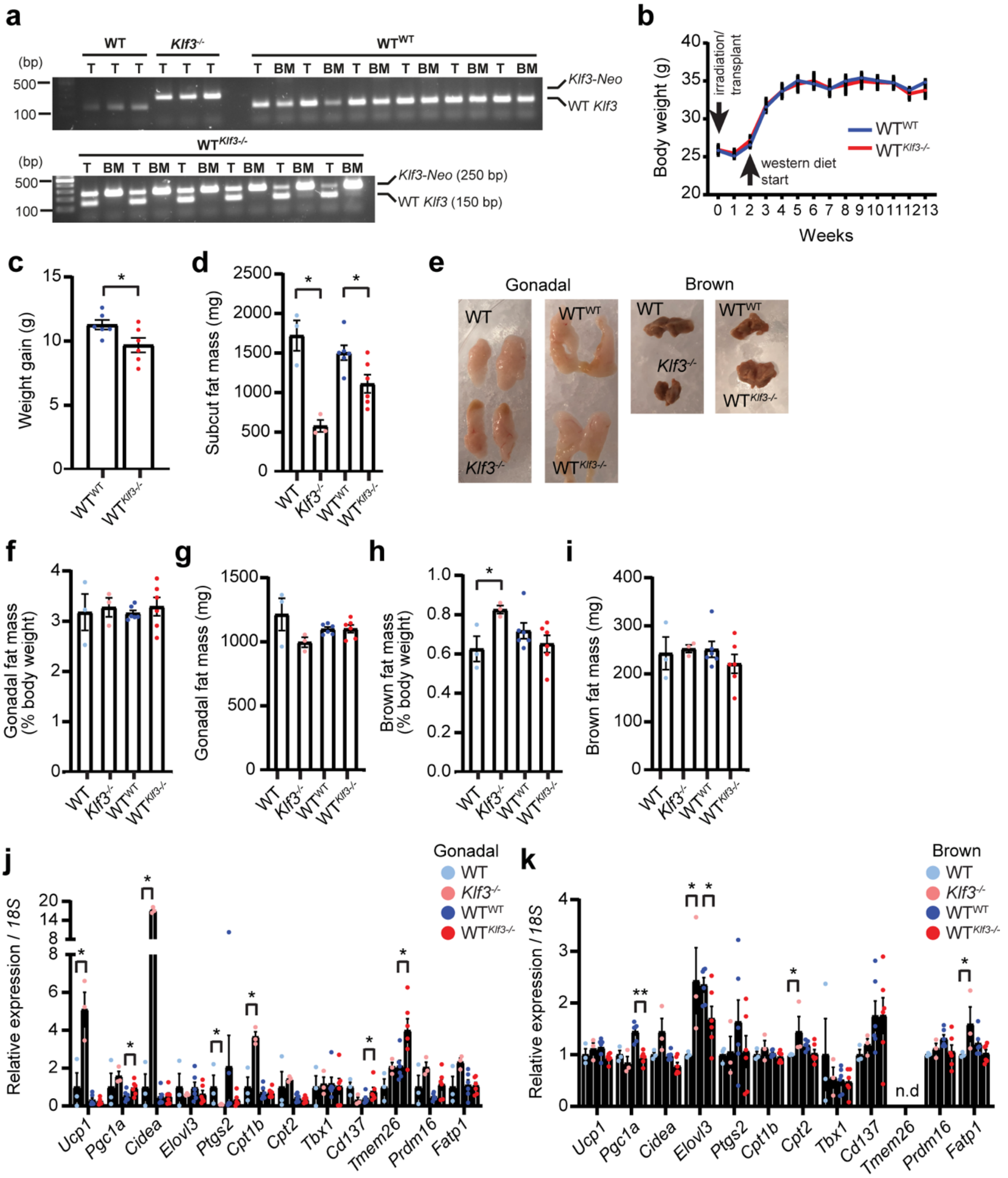

Supplementary Fig. 4. Related to Fig. 3. Increased beiging in WT mice transplanted with *Klf3*$^{-/-}$ bone marrow. a, genotyping of tail biopsies (T) and bone marrow (BM) was performed for WT, *Klf3*$^{-/-}$, WT^{WT} and WT$^{Klf3-/-}$ mice to determine irradiation success and transplant reconstitution (n=3-6 mice). A DNA ladder was loaded onto 2% agarose gels alongside samples. Lower bands represent WT *Klf3* (~150 bp) and upper bands represent the product of neomycin insertion into *Klf3* (*Klf3-Neo*; ~250 bp). **b**, body weight (g) in the 2 weeks following irradiation then over the duration of the Western diet study (weeks 2-13) for WT^{WT} and WT$^{Klf3-/-}$ mice was recorded weekly and **c**, body weight (g) gain calculated from the commencement of Western diet to the completion of the regimen (n=6 mice). **d**, absolute subcut AT mass (mg) was recorded for WT, *Klf3*$^{-/-}$, WT^{WT} and WT$^{Klf3-/-}$ mice at the end of the study. **e**, representative macroscopic images of gonadal and brown AT pads from WT, *Klf3*$^{-/-}$ WT^{WT} and WT$^{Klf3-/-}$ mice at the conclusion of the 11-week Western diet showing size and complexion. Mice had their **f**, gonadal (% body weight), **g**, gonadal (mg), **h**, brown (% body weight) and **i**, brown (mg) AT weighed at the conclusion of the study. mRNA levels of thermogenic genes in **j**, gonadal and **k**, brown AT were assessed by qPCR. Expression was normalised to levels of *18S* rRNA. For **d** and **f-k**, (n=3 WT, *Klf3*$^{-/-}$ and n=6 WT^{WT} and WT$^{Klf3-/-}$ mice). For **b-d** and **f-k**, error bars represent means ± SEM and one-sided non-parametric Mann-Whitney U tests were performed where *P<0.05. Source data are provided as a Source Data file. bp, base pairs.

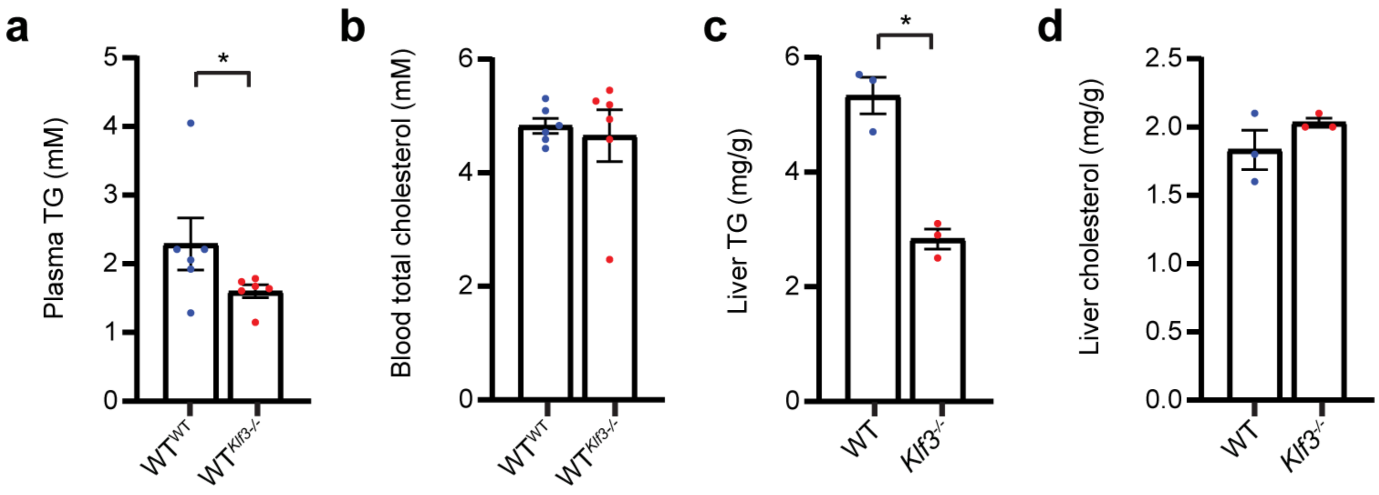

Supplementary Fig. 5. Related to Fig. 3. Increased beiging in WT mice transplanted with *Klf3*$^{-/-}$ bone marrow and evidence of additional metabolic effects in this mouse model. Plasma from WT^{WT} and WT$^{Klf3-/-}$ mice fed a Western diet was analysed for levels of **a**, triglycerides and **b**, total cholesterol (n=6 mice). Hepatic **c**, triglyceride and **d**, cholesterol content was also tested in WT and *Klf3*$^{-/-}$ untransplanted mice fed a chow diet (n=3 mice). Concentration was normalised to liver weight. For **a-d**, error bars represent the means ± SEM and one-sided non-parametric Mann-Whitney U tests were performed where *P<0.05. Source data are provided as a Source Data file.

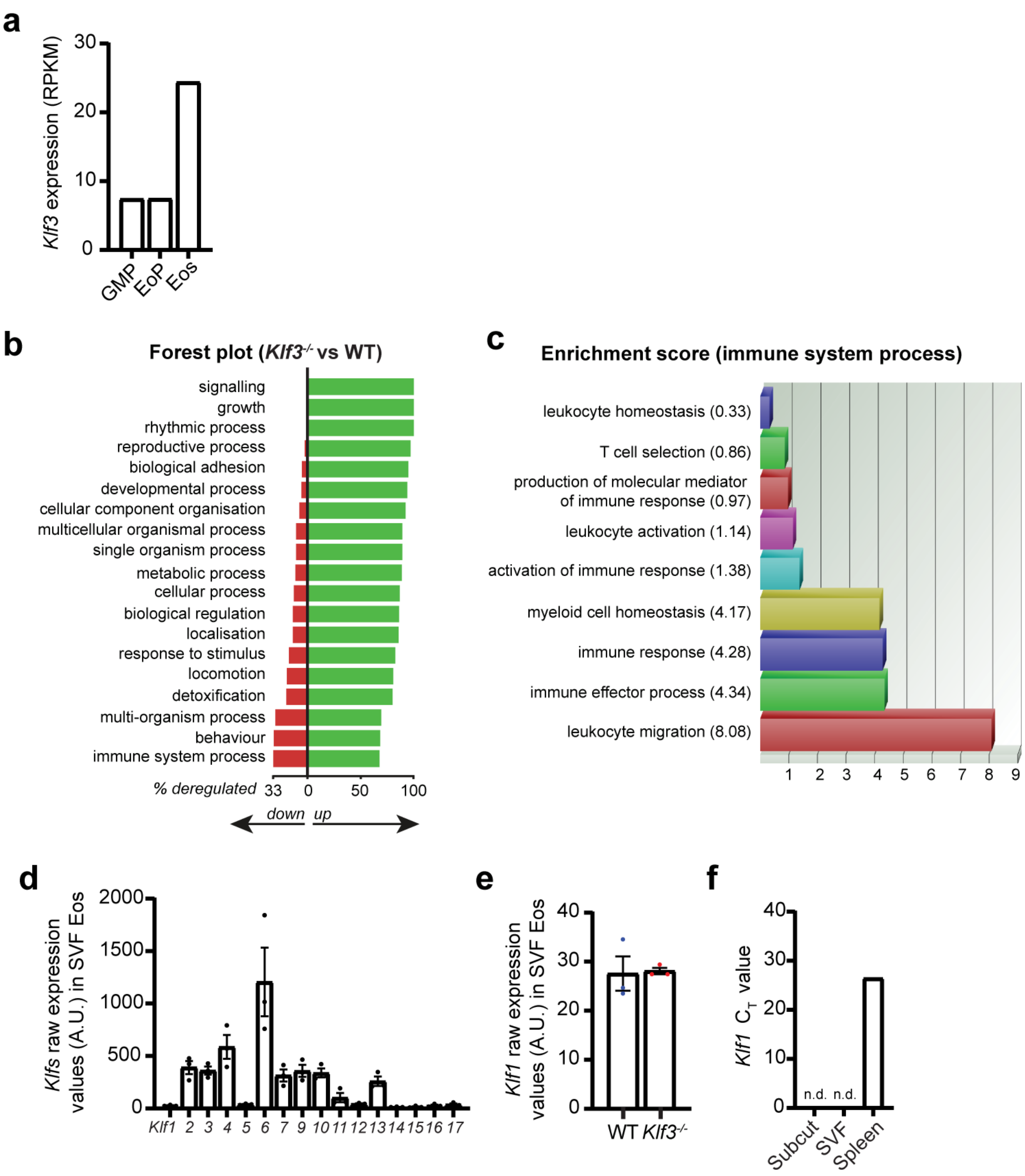

Supplementary Fig. 6. Related to Fig. 4. AT-resident eosinophils are altered in the absence of KLF3. a, *Klf3* was identified as highly expressed in a transcription factor screen of eosinophil lineage populations[1]. Expression is represented as RNA-Seq reads per kilobase million (RPKM) for granulocyte-macrophage progenitors (GMP), eosinophil progenitors (EoP) and mature eosinophils (Eos). **b**, gene ontology (GO) analysis of the significantly deregulated genes (*Klf3*$^{-/-}$ vs WT; $P<0.05$ using Fisher's Exact test) identified in subcut SVF eosinophil microarrays was performed using Partek Genomics Suite software and a forest plot constructed showing key implicated cellular processes. GO terms are listed by the number of significantly up-regulated genes, with the percent up-regulated and down-regulated shown in red to green bars. Multiple comparisons adjustments were not made. **c**, enrichment of deregulated biological pathways within immune system processes (from **b**) represented as a bar plot, generated using Partek Genomics Suite software. **d**, Raw expression values for all *Klf* family members from mouse SVF eosinophil microarrays (n=3 WT mice). Error bars are means ± SEM. No probe ID exists for *Klf8* in the array chip used. **e**, Raw expression values for *Klf1* in WT and *Klf3*$^{-/-}$ SVF eosinophils (n=3 mice). Error bars are means ± SEM. **f**, C_T values for *Klf1* in whole subcut, subcut SVF and spleen from WT mice. For **e**, a one-sided non-parametric Mann-Whitney U test was performed to assess significance. Source data are provided as a Source Data file. A.U.; arbitrary units; n.d., not detected.

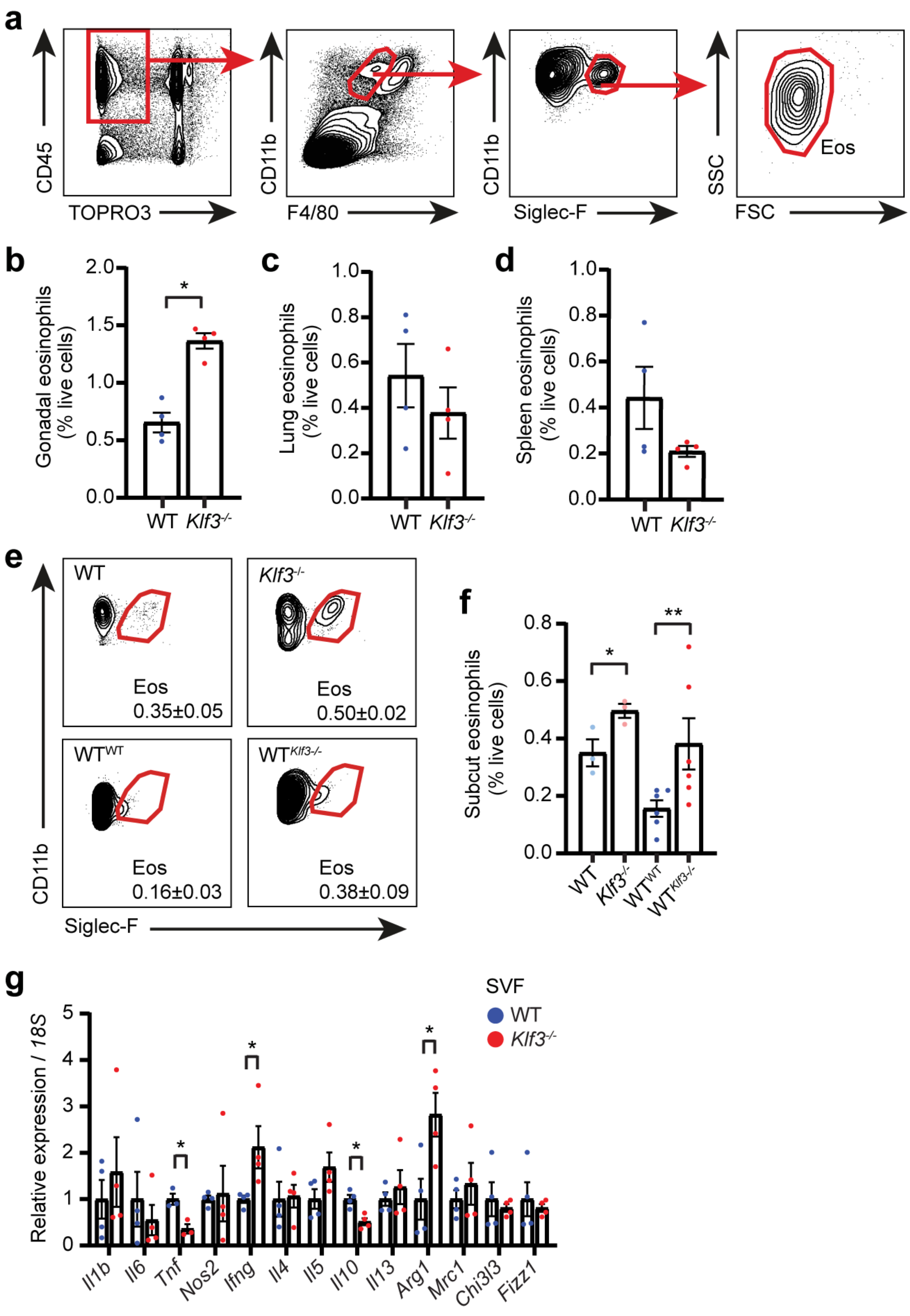

Supplementary Fig. 7. Related to Fig. 4. AT-resident eosinophils are altered in the absence of KLF3. a, gating strategy for identification of Siglec-F$^+$ eosinophils by flow cytometry (live CD45$^+$ CD11b$^+$ F4/80$^+$ Siglec-F$^+$ SSC^{hi}). This gating strategy was used in Fig. 4a, b and Supplementary Fig. 7b-f. Siglec-F$^+$ eosinophils were assessed as a percentage of live cells in WT and *Klf3*$^{-/-}$ **b**, gonadal SVF, **c**, lung and **d**, spleen (n=4 mice). **e**, flow cytometry plots showing abundance of Siglec-F$^+$ eosinophils (% of live cells) in the subcut SVF of WT, *Klf3*$^{-/-}$, WT^{WT} and WT$^{Klf3-/-}$ mice fed a Western diet for 11 weeks, including means ± SEM. Graphical representation is shown in **f**, (n=3 WT, *Klf3*$^{-/-}$ and n=6 WT^{WT} and WT$^{Klf3-/-}$ mice). **g**, mRNA levels of inflammatory and anti-inflammatory marker genes in WT and *Klf3*$^{-/-}$ subcut SVF were assessed by qPCR. Relative expression was normalised to levels of *18S* rRNA (n=4 mice). For **b-d** and **f-g**, error bars represent means ± SEM and one-sided non-parametric Mann-Whitney U tests were performed where *P<0.05, **P<0.01. Source data are provided as a Source Data file. FSC, forward scatter; SSC, side scatter; Eos, eosinophils.

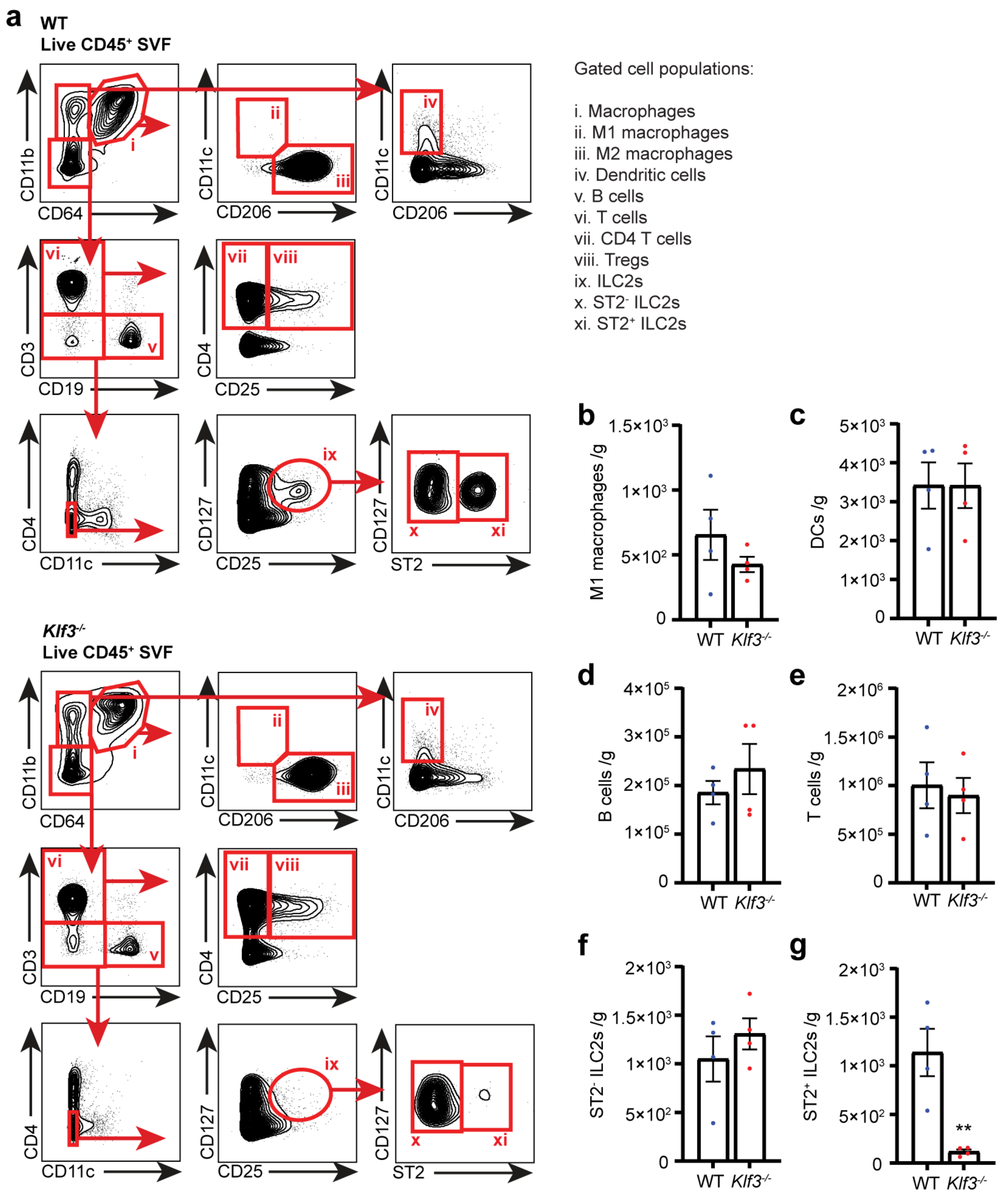

Supplementary Fig. 8. Related to Fig. 4. AT-resident eosinophils are altered in the absence of KLF3. a, Gating strategy for adipose immunophenotyping in representative WT and *Klf3*$^{-/-}$ subcut AT SVF. This strategy was used in Fig. 4g-j and Supplementary Fig. 8b-g. Gated populations of live CD45^{+} cells were defined as (i) macrophages (CD11b^{+} CD64^{+}), (ii) M1 macrophages (CD11b^{+} CD64^{+} CD11c^{+} CD206^{-}), (iii) M2 macrophages (CD11b^{+} CD64^{+} CD11c^{-} CD206^{+}), (iv) dendritic cells (CD11b^{+} CD64^{-} CD11c^{+}), (v) B cells (CD11b^{-} CD64^{-} CD19^{+} CD3^{-}), (vi) T cells (CD11b^{-} CD64^{-} CD19^{-} CD3^{+}), (vii) CD4 T cells (CD11b^{-} CD64^{-} CD19^{-} CD3^{+} CD4^{+} CD25^{-}), (viii) Tregs (CD11b^{-} CD64^{-} CD19^{-} CD3^{+} CD4^{+} CD25^{+}), (ix) ILC2s (CD11b^{-} CD64^{-} CD19^{-} CD3^{-} CD4^{-} CD11c^{-} CD127^{+} CD25^{+}) which were further divided into (x) ST2^{-} and (xi) ST2^{+} ILC2s. Abundance of subcut AT-resident **b**, M1 macrophages, **c**, dendritic cells (DCs), **d**, B cells, **e**, T cells, **f**, ST2^{-} and **g**, ST2^{+} ILC2s were quantified and normalized to tissue weight (n=4 mice). For **b-g**, error bars represent means ± SEM and one-sided non-parametric Mann-Whitney U tests were performed where *P<0.05, **P<0.01. Source data are provided as a Source Data file.

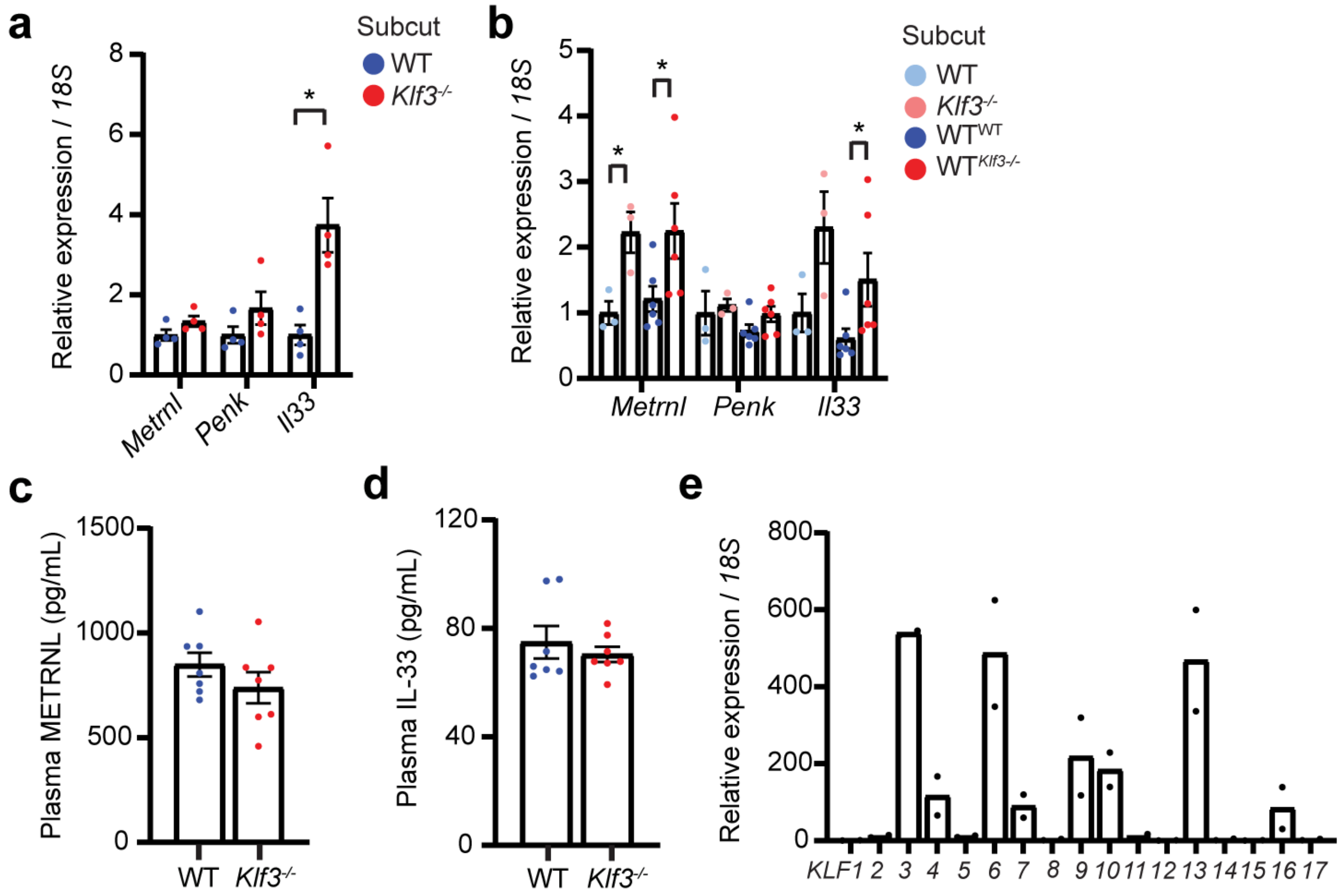

Supplementary Fig. 9. Related to Fig. 5. KLF3 regulates AT eosinophil gene expression. qPCR was used to assess mRNA levels of *Metrnl*, *Penk* and *Il33* in subcut AT from **a**, WT and *Klf3*$^{-/-}$ mice on a chow diet (n=4 mice), and from **b**, WT, *Klf3*$^{-/}$ (n=3)$^{-}$, WT^{WT} and WT$^{Klf3-/-}$ (n=6) mice following an 11-week Western diet. Relative expression was normalised to levels of *18S* rRNA and the WT value for each gene set to 1. Circulating levels of **c**, meteorin-like and **d**, IL-33 were analysed by ELISA in plasma from WT and *Klf3*$^{-/-}$ mice (n=7 mice). **e**, mRNA levels of all *KLF* family member genes were assessed by qPCR in EoL-1 cells to determine relative expression of *KLF3* normalised to levels of *18S* rRNA. Bars represent the means of n=2 replicates. For **a-d**, error bars represent means ± SEM and one-sided non-parametric Mann-Whitney U tests were performed where *P<0.05. Source data are provided as a Source Data file.

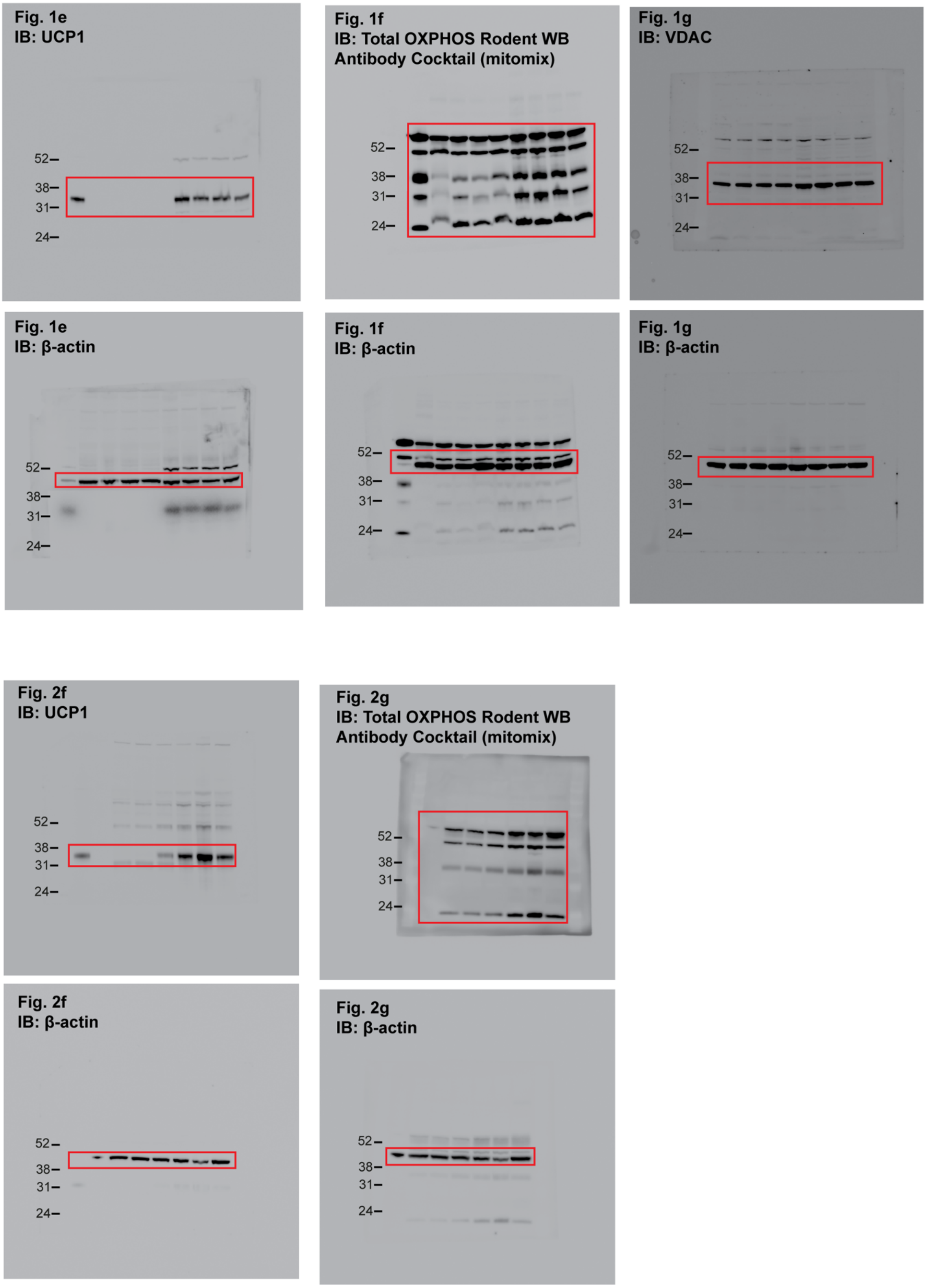

Supplementary Fig. 10. Western blot full scans.

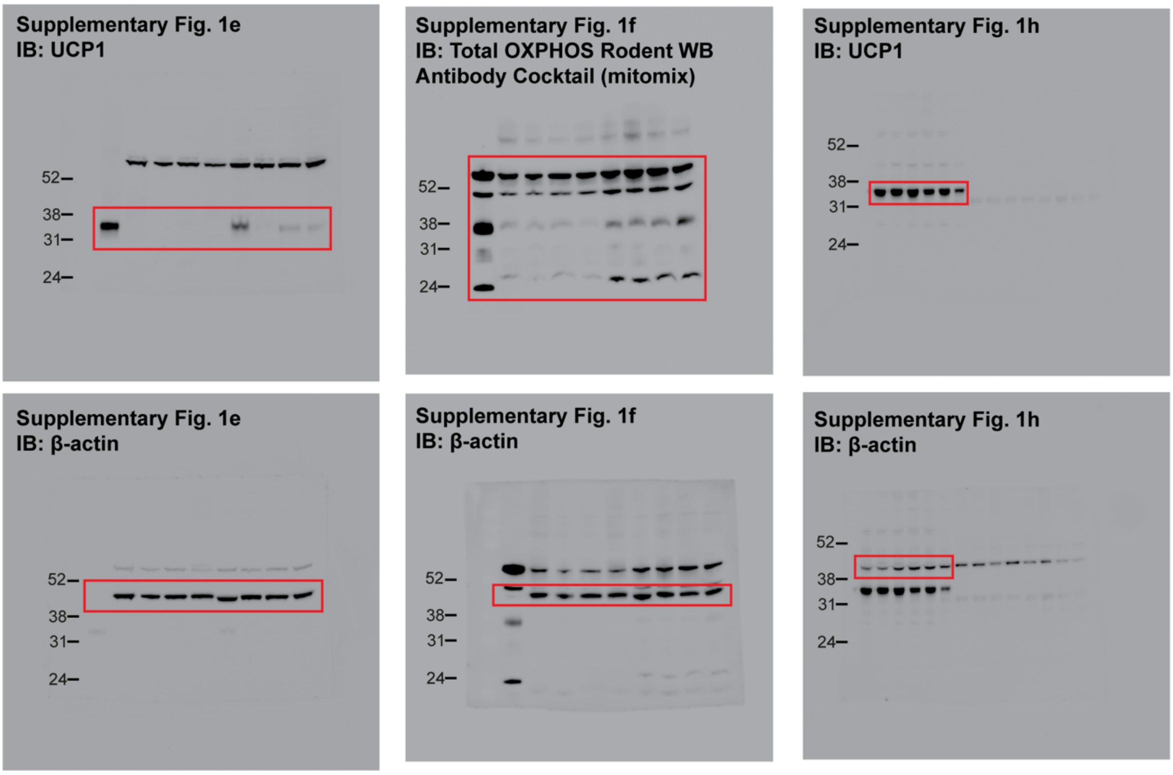

(Continued) Supplementary Fig. 10. Western blot full scans.

Supplementary Table 1. Mouse diets.

Diet	Ingredients	Caloric breakdown (kCal %)
Chow<br>*Gordon's Specialty Feeds*	Wheat<br>Sorghum<br>Soybean meal<br>Pollard<br>Bran<br>Meat and bone meal<br>Bloodmeal<br>Fish meal<br>Lucerne meal<br>Vegetable oil<br>Sunflower meal<br>Salt<br>Vitamin and mineral premix<br>Lysine<br>Choline chloride	Carbohydrate 58%<br>Fat 16%<br>Protein 26%
Western (high-fat, high-sugar)<br>*Homemade*	Sucrose<br>Corn starch<br>Wheat bran<br>Casein<br>Methionine<br>Gelatine<br>Choline bitartate<br>Lard<br>Safflower oil<br>AIN-93M mineral mix<br>AIN-93-VX vitamin mix<br>Trace mineral mix	Carbohydrate 36%<br>Fat 45%<br>Protein 19%

Supplementary Table 2. Mouse and human oligonucleotides.

Name	Sequence	Application
WT *Klf3* geno Fwd	GGCAAATCTGGCGCATCTGA	Mouse genotyping
WT *Klf3* geno Rev	TGGCTGGGAGCCCCTCTCAT	Mouse genotyping
Klf3-Neo geno Fwd	TCCATGTCTGTCTCCCCCTA	Mouse genotyping
Klf3-Neo geno Rev	ATTAAGGGCCAGCTCATTCC	Mouse genotyping
18S rRNA Fwd	CACGGCCGGTACAGTGAAAC	qPCR (mouse and human)
18S rRNA Rev	AGAGGAGCGAGCGACCAA	qPCR (mouse and human)
mUcp1 Fwd	GGAGAGAAACACCTGCCTCT	qPCR (mouse)
mUcp1 Rev	CTGACCTTCACGACCTCTGT	qPCR (mouse)
mPgc1a Fwd	TGAAGACGGATTGCCCTCAT	qPCR (mouse)
mPgc1a Rev	TGGTGCCAGTAAGAGCTTCT	qPCR (mouse)
mCidea Fwd	TGGTGGACACAGAGGAGTTC	qPCR (mouse)
mCidea Rev	GTGACTCTGGCTATTCCCGA	qPCR (mouse)
mElovl3 Fwd	TGGTCCTTCTTCCTGGCAAT	qPCR (mouse)
mElovl3 Rev	TGTAGATGGCAAAGCACACG	qPCR (mouse)
mPtgs2 Fwd	GAACAACATCCCCTTCCTGC	qPCR (mouse)
mPtgs2 Rev	AGAGGTTGGAGAAGGCTTCC	qPCR (mouse)
mCpt1b Fwd	GCAGGAGGAAGGGTAGAGTG	qPCR (mouse)
mCpt1b Rev	GGGTCACAAAGAAAGCAGCA	qPCR (mouse)
mCpt2 Fwd	ACAGCCAGTTCAGGAAGACA	qPCR (mouse)
mCpt2 Rev	CTGAGATGTAGCTGGTGTGC	qPCR (mouse)
mTbx1 Fwd	CGCACAGTGGATGAAACAGA	qPCR (mouse)
mTbx1 Rev	TGTCTTTTCGAGGGTCCACA	qPCR (mouse)
mCd137 Fwd	CGTGCAGAACTCCTGTGATAAC	qPCR (mouse)
mCd137 Rev	GTCCACCTATGCTGGAGAAGG	qPCR (mouse)
mTmem26 Fwd	CGGCCATCTTTGTGTACCTG	qPCR (mouse)
mTmem26 Rev	TGTGGGATGACAGGGTTTGA	qPCR (mouse)
mPrdm16 Fwd	ATGACCAAAACCTCGCCATG	qPCR (mouse)
mPrdm16 Rev	ATGACACCCAAGGAGTAGGC	qPCR (mouse)
mFatp1 Fwd	GTTCTGTGTGTACGTGGGTG	qPCR (mouse)
mFatp1 Rev	GCCGAACACGAATCAGAACA	qPCR (mouse)
mSiglecf Fwd	CAGGGACGTACTTCTTCAGATTGG	qPCR (mouse)
mSiglecf Rev	GGGTAGATGTGACTTGGATGTTAGG	qPCR (mouse)
mMetrnl Fwd	CTGGAGCAGGGAGGCTTATTT	qPCR (mouse)
mMetrnl Rev	GGACAACAAAGTCACTGGTACAG	qPCR (mouse)
mPenk Fwd	GCCAGGACTGCGCTAAATG	qPCR (mouse)
mPenk Rev	CCTTGCAGGTCTCCCAGATT	qPCR (mouse)
mIl33 Fwd	ACTTCTCTGCCTATCCACGG	qPCR (mouse)
mIl33 Rev	GTGAAGTTCCTTGGATGCTCA	qPCR (mouse)
mIl1b Fwd	TGTAATGAAAGACGGCACACC	qPCR (mouse)
mIl1b Rev	TCTTCTTTGGGTATTGCTTGG	qPCR (mouse)
mIl6 Fwd	AAAGCCAGAGTCCTTCAGAGAGATAC	qPCR (mouse)

mIl6 Rev	CTGTTAGGAGAGCATTGGAAATTG	qPCR (mouse)
mTnf Fwd	GCCACCACGCTCTTCTGTCT	qPCR (mouse)
mTnf Rev	GCCATAGAACTGATGA	qPCR (mouse)
mNos2 Fwd	CAGAGGACCCAGAGACAAGC	qPCR (mouse)
mNos2 Rev	TGCTGAAACATTTCCTGTGC	qPCR (mouse)
mIfng Fwd	TTTGCAGCTCTTCCTCATGG	qPCR (mouse)
mIfng Rev	TCCACATCTATGCCACTTGAGT	qPCR (mouse)
mIl4 Fwd	CAGCAACGAAGAACACCACA	qPCR (mouse)
mIl4 Rev	TCTGCAGCTCCATGAGAACA	qPCR (mouse)
mIl5 Fwd	GGAGATTCCCATGAGCACAG	qPCR (mouse)
mIl5 Rev	TAGGGACAGGAAGCCTCATC	qPCR (mouse)
mIl10 Fwd	AGTACAGCCGGGAAGACAAT	qPCR (mouse)
mIl10 Rev	AGCTGGTCCTTTGTTTGAAAGA	qPCR (mouse)
mIl13 Fwd	CTGTGTCTCTCCCTCTGACC	qPCR (mouse)
mIl13 Rev	CACACTCCATACCATGCTGC	qPCR (mouse)
mArg1 Fwd	CTCCAAGCCAAAGTCCTTAGAG	qPCR (mouse)
mArg1 Rev	AGGAGCTGTCATTAGGGACATC	qPCR (mouse)
mMrc1 Fwd	CATGAGGCTTCTCCTGTTCT	qPCR (mouse)
mMrc1 Rev	TTGCCGTCTGAACTGAGATGG	qPCR (mouse)
mChi3l3 Fwd	AGAAGGGAGTTTCAAACCTGGT	qPCR (mouse)
mChi3l3 Rev	GTCTTGCTCATGTGTGTAAGTGA	qPCR (mouse)
mFizz1 Fwd	TACTTGCAACTGCCTGTGCTTACT	qPCR (mouse)
mFizz1 Rev	TATCAAAGCTGGGTTCTCCACCTC	qPCR (mouse)
mKlf1 Fwd	AGACTGTCTTACCCTCCATCAGTACA	qPCR (mouse)
mKlf1 Rev	CCGCCACCACTTGAGGAA	qPCR (mouse)
hKLF1 Fwd	GGTTGCGGCAAGAGCTACA	qPCR (human)
hKLF1 Rev	ACACAGGGGAGAAGCCATACG	qPCR (human)
hKLF2 Fwd	AGGTCGTCGTCGGTGC	qPCR (human)
hKLF2 Rev	TGCCGTCCTTCTCCACTTTCG	qPCR (human)
hKLF3 Fwd	ACCCAGTTCCTGTCAAGCAA	qPCR (human)
hKLF3 Rev	TCAGGCAATGGTGTGGAGTA	qPCR (human)
hKLF4 Fwd	CATTACCAAGAGCTCATGCCACC	qPCR (human)
hKLF4 Rev	CAGCCCGCGTAATCACAAG	qPCR (human)
hKLF5 Fwd	TTTAAAAGCTCACCTGAGGACTCA	qPCR (human)
hKLF5 Rev	CAGCCTTCCCAGGTACACTTG	qPCR (human)
hKLF6 Fwd	TCTGGAGGAGTACTGGCAACAG	qPCR (human)
hKLF6 Rev	GCTCGCTCTGGAGGTAACGT	qPCR (human)
hKLF7 Fwd	CCGGCTACTTCTCAGCTTTACC	qPCR (human)
hKLF7 Rev	CGTTCCAATTCAAGGCATGTC	qPCR (human)
hKLF8 Fwd	ACCAAAAGCTCTCACCTGAAAGC	qPCR (human)
hKLF8 Rev	TCTGAGCGAGCAAATTTCCA	qPCR (human)
hKLF9 Fwd	CCATTACAGAGTGCATACAGGTGAA	qPCR (human)
hKLF9 Rev	TGAGCGGGAGAACTTTTTAAGG	qPCR (human)

hKLF10 Fwd	GGTGCCTCTCTCCAGCAGACT	qPCR (human)
hKLF10 Rev	CTTTTGGCCTTTCAGAAATCATTT	qPCR (human)
hKLF11 Fwd	AGCCACACCTGAACTACCAAAAG	qPCR (human)
hKLF11 Rev	GGCTCTGAGGAGGAGTTATGCA	qPCR (human)
hKLF12 Fwd	AAAACAGAGCTTTTGGAATCTGAAC	qPCR (human)
hKLF12 Rev	GCTTCCATATCGGGATAGTTGTG	qPCR (human)
hKLF13 Fwd	GAAATCTTCGCACCTCAAG	qPCR (human)
hKLF13 Rev	GAACTTCTTGTTGCAGTCCT	qPCR (human)
hKLF14 Fwd	CCGGAGGAGGTCTGTCACA	qPCR (human)
hKLF14 Rev	TGCGACGACTTGTAATAGGCTTT	qPCR (human)
hKLF15 Fwd	GACCGAGAGTCTAGCCG	qPCR (human)
hKLF15 Rev	TAAGTGGTCCACCATGCTG	qPCR (human)
hKLF16 Fwd	GCGCCAAAGCCTACTACAAGTC	qPCR (human)
hKLF16 Rev	TGCCAGTCACAAGCAAAAGG	qPCR (human)
hKLF17 Fwd	CCAGGCTGCCCAGGATAA	qPCR (human)
hKLF17 Rev	CTTCCAGAAGATGAAGACATGTTCA	qPCR (human)
hSP1 ChIP Fwd	ACCTCTCCGCCCACTAGGA	ChIP-qPCR (human)
hSP1 ChIP Rev	CAACGGCCAACCAGAATCC	ChIP-qPCR (human)
hMETRNL 1 ChIP Fwd	AGGAACACACTCGAGCAGG	ChIP-qPCR (human)
hMETRNL 1 ChIP Rev	CCCCAGCTTTGACAACCTAA	ChIP-qPCR (human)
hMETRNL 2 ChIP Fwd	CCCTGACCCAGTTTCTCTTATG	ChIP-qPCR (human)
hMETRNL 2 ChIP Rev	CTGATGGAGGCTTGAGACATT	ChIP-qPCR (human)
hPENK ChIP Fwd	CGCGGTTCCTGACACTTT	ChIP-qPCR (human)
hPENK ChIP Rev	GCACTAGGCGGTAGCTG	ChIP-qPCR (human)
hIL33 1 ChIP Fwd	GCAGTGCAGAGTACCATATCC	ChIP-qPCR (human)
hIL33 1 ChIP Rev	GTGGCTAGCTCACTACCAAAT	ChIP-qPCR (human)
hIL33 2 ChIP Fwd	GCACAAAGGCGTTCAACAA	ChIP-qPCR (human)
hIL33 2 ChIP Rev	TCTCTGGAATGTGTCTGTGTTC	ChIP-qPCR (human)
hVEGFA ChIP Fwd	GGTTTGTATCCTGCCCTTCC	ChIP-qPCR (human)
hVEGFA ChIP Rev	ACTGGGTCTTGCTGTTTTCC	ChIP-qPCR (human)
hKLF3 sgRNA Fwd	CACCGAACTCCGCTTGTTCACCGTG	sgRNA oligo for CRISPR
hKLF3 sgRNA Rev	AAACCACGGTGAACAAGCGGAGTTC	sgRNA oligo for CRISPR
hKLF3 ex3 geno PCR	CTTATTTGGCTGTTGACACG	CRISPR clone screening
hKLF3 ex3 geno PCR Rev	GGCAGATGAGTATTTCCTTTG	CRISPR clone screening

Supplementary Table 3. Antibodies

Antibody name	Raised in	Supplier/Product#	Application
Mouse polyclonal anti-UCP1	Rabbit	Abcam ab10983	Western blotting (1:1,000)
Mouse monoclonal anti-VDAC	Rabbit	Cell Signaling 4661	Western blotting (1:1,000)
Mouse monoclonal anti-β-actin clone AC-15	Mouse	Sigma A1978	Western blotting (1:20,000)
Total OXPHOS Rodent Antibody Cocktail	Mouse	Abcam ab110413	Western blotting (1:1,000)
ECL™ HRP-linked anti-mouse IgG		GE Healthcare NA931	Western blotting (1:10,000-20,000)
ECL™ HRP-linked anti-rabbit IgG		GE Healthcare NA934	Western blotting (1:10,000)
Mouse/human polyclonal anti-KLF3	Goat	Thermo Fisher Pierce PA5-18030	ChIP (15 μg per IP)
Normal goat IgG		Santa Cruz Biotechnology SC-2028	ChIP (15 μg per IP)
Anti-mouse CD16/32 Fc block clone 2.4G2	Rat	BD Pharmingen 553141	Flow cytometry (1:25)
BV421 anti-mouse Siglec-F clone E50-2440	Rat	BD Horizon 562681	Flow cytometry (1:33)
Biotin anti-mouse CD45 clone 30-F11	Rat	BD Pharmingen 553078	Flow cytometry (1:20)
BV711 Streptavidin		BD Horizon 563262	Flow cytometry (1:100)
FITC anti-mouse CD11b clone M1/70	Rat	BD Pharmingen 557396	Flow cytometry (1:100)
PE/Cy5 anti-mouse F4/80 clone BM8	Rat	eBioscience 15-4801-80	Flow cytometry (1:33)
PE/Cy7 anti-mouse F4/80 clone BM8	Rat	Biolegend 123113	Flow cytometry (1:33)
PE/Cy7 anti-mouse CD25 clone PC61	Rat	BD Pharmingen 552880	Flow cytometry (1:33)
APC/Cy7 anti-mouse TCRβ clone H57-597	Hamster	Biolegend 109220	Flow cytometry (1:100)
BUV395 anti-mouse CD45 clone 30-F11	Rat	BD Horizon 564279	Flow cytometry (1:50)
BV421 anti-mouse CD206 clone C068C2	Rat	Biolegend 141717	Flow cytometry (1:50)
BV510 anti-mouse NK1.1 clone PK-136	Rat	BD Horizon 563096	Flow cytometry (1:25)
BV605 anti-mouse CD64 clone X54-5/7.1	Mouse	Biolegend 139323	Flow cytometry (1:20)
BV650 anti-mouse CD11b clone M1/70	Rat	BD Horizon 563402	Flow cytometry (1:100)
BV711 anti-mouse CD11c clone HL3	Hamster	BD Horizon 563048	Flow cytometry (1:20)
BV786 anti-mouse CD19 clone 1D3	Rat	BD Horizon 563333	Flow cytometry (1:50)
FITC anti-mouse CD3e clone 145-2C11	Hamster	BD Pharmingen 561827	Flow cytometry (1:100)
PE anti-mouse IL-33R/ST2 clone U29-93	Rat	BD Pharmingen 566312	Flow cytometry (1:20)
AF700 anti-mouse CD4 clone RM4-5	Rat	BD Pharmingen 557956	Flow cytometry (1:50)

Supplementary References

1. Bouffi C*, et al.* Transcription Factor Repertoire of Homeostatic Eosinophilopoiesis. *J Immunol* **195**, 2683-2695 (2015).
