# Supplementary material for: Eosinophil function in adipose tissue is regulated by Krüppel-like factor 3 (KLF3)
Source: Nat Commun. 2020 Jun 10;11:2922. doi: 10.1038/s41467-020-16758-9 (PMC7286919; doi:10.1038/s41467-020-16758-9)
Supplement: Supplementary file 2 — Reporting Summary [file 41467_2020_16758_MOESM2_ESM.pdf]

nature research

Corresponding author(s): Kate G. R. Quinlan

# Reporting Summary

Nature Research wishes to improve the reproducibility of the work that we publish. This form provides structure for consistency and transparency in reporting. For further information on Nature Research policies, see Authors & Referees and the Editorial Policy Checklist.

## Statistical parameters

When statistical analyses are reported, confirm that the following items are present in the relevant location (e.g. figure legend, table legend, main text, or Methods section).

| n/a | Confirmed | |
|---|---|---|
| ☐ | ☒ | The exact sample size ($n$) for each experimental group/condition, given as a discrete number and unit of measurement |
| ☐ | ☒ | An indication of whether measurements were taken from distinct samples or whether the same sample was measured repeatedly |
| ☐ | ☒ | The statistical test(s) used AND whether they are one- or two-sided<br>*Only common tests should be described solely by name; describe more complex techniques in the Methods section.* |
| ☐ | ☒ | A description of all covariates tested |
| ☐ | ☒ | A description of any assumptions or corrections, such as tests of normality and adjustment for multiple comparisons |
| ☐ | ☒ | A full description of the statistics including central tendency (e.g. means) or other basic estimates (e.g. regression coefficient) AND variation (e.g. standard deviation) or associated estimates of uncertainty (e.g. confidence intervals) |
| ☐ | ☒ | For null hypothesis testing, the test statistic (e.g. $F$, $t$, $r$) with confidence intervals, effect sizes, degrees of freedom and $P$ value noted<br>*Give P values as exact values whenever suitable.* |
| ☒ | ☐ | For Bayesian analysis, information on the choice of priors and Markov chain Monte Carlo settings |
| ☒ | ☐ | For hierarchical and complex designs, identification of the appropriate level for tests and full reporting of outcomes |
| ☒ | ☐ | Estimates of effect sizes (e.g. Cohen's $d$, Pearson's $r$), indicating how they were calculated |
| ☐ | ☒ | Clearly defined error bars<br>*State explicitly what error bars represent (e.g. SD, SE, CI)* |

*Our web collection on statistics for biologists may be useful.*

## Software and code

Policy information about availability of computer code

| | |
|---|---|
| Data collection | No software was used for data collection |
| Data analysis | Partek Genomics Suite version 7 (microarray analysis), GraphPad Prism version 8 (generation of graphs and statistical tests), Adobe Illustrator CS5.1 (compilation of figures), Adobe Photoshop CS5.1 (Western blots), FlowJo version 10 (flow cytometry and FACS analysis), Applied Biosystems 7500 version 2.3 and QuantStudio Real-Time PCR version 1.3 software (qPCR analysis), primer3 version 4.1.0 online tool (primer design), Integrative Genomics Viewer version 2.4.9 (Broad Institute), Bio-Rad Image Lab version 6.0.1 software (agarose gel analysis), Optimized CRISPR design version 1 online tool (sgRNA design), Aperio ImageScope (processing and analysis of H&E staining), BioRender (creating licensed cartoons/figures) |

For manuscripts utilizing custom algorithms or software that are central to the research but not yet described in published literature, software must be made available to editors/reviewers upon request. We strongly encourage code deposition in a community repository (e.g. GitHub). See the Nature Research guidelines for submitting code & software for further information.

## Data

Policy information about availability of data

All manuscripts must include a data availability statement. This statement should provide the following information, where applicable:

- Accession codes, unique identifiers, or web links for publicly available datasets
- A list of figures that have associated raw data
- A description of any restrictions on data availability

Data supporting the findings of this study can be found within the paper and its Supplementary Information files, and a Source Data file has been provided for data underlying figures. Further information is available upon reasonable request. Full scans of Western blots are available in Supplementary Fig. 10. Datasets are publically available from the NCBI Gene Expression Omnibus (GEO) using Accession Nos. GSE117445 (https://www.ncbi.nlm.nih.gov/geo/query/acc.cgi?acc=GSE117445) and GSE44748 (https://www.ncbi.nlm.nih.gov/geo/query/acc.cgi?acc=GSE44748), FANTOM5 SSTAR (https://fantom.gsc.riken.jp/5/sstar/Main_Page) and Haemosphere (https://www.haemosphere.org/). A reporting summary has also been provided as part of the Supplementary Information files.

# Field-specific reporting

Please select the best fit for your research. If you are not sure, read the appropriate sections before making your selection.

[x] Life sciences [ ] Behavioural & social sciences [ ] Ecological, evolutionary & environmental sciences

For a reference copy of the document with all sections, see nature.com/authors/policies/ReportingSummary-flat.pdf

# Life sciences study design

All studies must disclose on these points even when the disclosure is negative.

| | |
|---|---|
| Sample size | For animal experiments, sample sizes were determined based on previous experimentation with this animal model (Knights et al, J Biol Chem, 2020, doi: 10.1074/jbc.RA120.013114; Knights et al, J Biol Chem, 2016, doi: 10.1074/jbc.M116.715748; Bell-Anderson et al, Diabetes, 2013, doi: 10.2337/db12-1745), with numbers selected as those deemed necessary to observe phenotypic and genetic changes with statistical significance while reducing animal overconsumption.<br>For EoL-1 ChIP-qPCR experiments, sample size was determined based on previous experience with this technique in cell lines (Martyn et al, Nat Genet, 2018, doi: 10.1038/s41588-018-0085-0; Wienert et al, Blood, 2017, doi: 10.1182/blood-2017-02-767400; Lim et al, Nucleic Acids Res, 2016, doi: 10.1093/nar/gkv1380). |
| Data exclusions | No data exclusions |
| Replication | Results were replicated in multiple biological replicates (n=3 or more) using paired mice (sex and litter-matched) for all relevant experiments. For cell culture experiments, results were replicated across three experiments for ChIP-qPCR and two experiments for EoL-1 KLF gene expression, to ensure reproducibility. |
| Randomization | Randomisation was not relevant to this study as mouse experiments were performed on paired littermate mice (a WT and KLF3-/- littermate pair) which were matched for both age and gender. The paired mice were treated concurrently to ensure that covariates were appropriately controlled. For acute cold experiments littermate (WT and KLF3-/-) mice were randomly allocated to either cold or thermoneutral groups. For bone marrow transplant recipients (all WT mice), mice were divided into cohorts of equal mean weights/body mass composition for receiving WT or KLF3 KO bone marrow to ensure that these groups had the same starting body composition prior to the Western diet feeding study. |
| Blinding | Investigators were not blinded to sample identity as all data produced was from objective quantitative methods so subjective bias was not relevant. |

# Reporting for specific materials, systems and methods

### Materials & experimental systems

| n/a | Involved in the study | |
|---|---|---|
| x | | Unique biological materials |
| | x | Antibodies |
| | x | Eukaryotic cell lines |
| x | | Palaeontology |
| | x | Animals and other organisms |
| x | | Human research participants |

### Methods

| n/a | Involved in the study | |
|---|---|---|
| x | | ChIP-seq |
| | x | Flow cytometry |
| x | | MRI-based neuroimaging |

# Antibodies

Antibodies used

Antibody name/clone -- Raised in -- Supplier/product# -- Application
-- ----

Mouse polyclonal anti-UCP1 Rabbit Abcam ab10983 Western blotting (1:1,000)
Mouse monoclonal anti-VDAC Rabbit Cell Signaling 4661 Western blotting (1:1,000)
Mouse monoclonal β-actin clone AC-15 Mouse Sigma A1978 Western blotting (1:20,000)
Total OXPHOS Rodent Antibody Cocktail Mouse Abcam ab110413 Western blotting (1:1,000)
ECL HRP-linked anti-mouse IgG GE Healthcare NA931 Western blotting (1:10,000-20,000)
ECL HRP-linked anti-rabbit IgG GE Healthcare NA934 Western blotting (1:10,000)
Mouse/human polyclonal anti-KLF3 Goat Thermo Fisher Pierce PA5-18030 ChIP (15 µg per IP)
Normal goat IgG Santa Cruz Biotechnology SC-2028 ChIP (15 µg per IP)
Anti-mouse CD16/32 Fc block clone 2.4G2 Rat BD Pharmingen 553141 Flow cytometry (1:25)
BV421 anti-mouse Siglec-F clone E50-2440 Rat BD Horizon 562681 Flow cytometry (1:33)
Biotin anti-mouse CD45 clone 30-F11 Rat BD Pharmingen 553078 Flow cytometry (1:20)
BV711 Streptavidin BD Horizon 563262 Flow cytometry (1:100)
FITC anti-mouse CD11b clone M1/70 Rat BD Pharmingen 557396 Flow cytometry (1:100)
PE/Cy5 anti-mouse F4/80 clone BM8 Rat eBioscience 15-4801-80 Flow cytometry (1:33)
PE/Cy7 anti-mouse F4/80 clone BM8 Rat Biolegend 123113 Flow cytometry (1:33)
PE/Cy7 anti-mouse CD25 clone PC61 Rat BD Pharmingen 552880 Flow cytometry (1:33)
APC/Cy7 anti-mouse TCRb clone H57-597 Hamster Biolegend 109220 Flow cytometry (1:100)
BUV395 anti-mouse CD45 clone 30-F11 Rat BD Horizon 564279 Flow cytometry (1:50)
BV421 anti-mouse CD206 clone C068C2 Rat Biolegend 141717 Flow cytometry (1:50)
BV510 anti-mouse NK1.1 clone PK-136 Rat BD Horizon 563096 Flow cytometry (1:25)
BV605 anti-mouse CD64 clone X54-5/7.1 Mouse Biolegend 139323 Flow cytometry (1:20)
BV650 anti-mouse CD11b clone M1/70 Rat BD Horizon 563402 Flow cytometry (1:100)
BV711 anti-mouse CD11c clone HL3 Hamster BD Horizon 563048 Flow cytometry (1:20)
BV786 anti-mouse CD19 clone 1D3 Rat BD Horizon 563333 Flow cytometry (1:50)
FITC anti-mouse CD3e clone 145-2C11 Hamster BD Pharmingen 561827 Flow cytometry (1:100)
PE anti-mouse IL-33R/ST2 clone U29-93 Rat BD Pharmingen 566312 Flow cytometry (1:20)
AF700 anti-mouse CD4 clone RM4-5 Rat BD Pharmingen 557956 Flow cytometry (1:50)

Validation

Antibody specificity for Western blotting was validated using protein extract from COS-7 cells lacking the mouse or human protein (as a negative control). Brown AT protein extract was used as a positive control for UCP1 due to high expression of this protein in this tissue type, and for mitochondrial proteins, rat heart mitochondria extract was provided with antibody cocktail as a positive control. For ChIP experiments, normal goat IgG was used as a negative control to the KLF3 IP, with the addition of input DNA that had not been immunoprecipitated. For flow cytometry, when designing panels, antibody titrations were performed to obtain optimal concentrations, with the addition of fluorescence-minus-one (FMO) controls to determine positive and negative population gating. To compensate for potential fluorescent spillover during experiments, single stained controls for each colour were performed using Invitrogen UltraComp eBeads (01-2222-41).

# Eukaryotic cell lines

Policy information about cell lines

Cell line source(s)

COS-7 (gift from Stuart Orkin, Harvard)
EoL-1 (ECACC; supplied by Sigma)

Authentication

No authentication was performed on COS-7 cells. ECACC performs cell line authentication on human cell lines (including EoL-1) by STR profiling

Mycoplasma contamination

ECACC ensures mycoplasma testing on all cell lines provided. Additionally, all cell lines used were subjected to mycoplasma testing services by the University of New South Wales School of Biotechnology and Biomolecular Sciences, and all lines were returned negative for contamination.

Commonly misidentified lines
(See ICLAC register)

No commonly misidentified cell lines were used

# Animals and other organisms

Policy information about studies involving animals; ARRIVE guidelines recommended for reporting animal research

Laboratory animals

Mus musculus, FVB/NJ, male, age ranged from 6-14 weeks. All animal work was carried out in accordance with approval from the UNSW Animal Care and Ethics Committee (Approval Nos. 12/150A, 16/5B and 16/141B), the Murdoch Children's Research Institute Animal Ethics Committee (Approval No. A760) and the University of Sydney Animal Care and Ethics Committee (Approval No. L02/7-2009/3/5054). Animals were housed in a specific pathogen-free environment at a constant ambient

temperature of 22°C, humidity of 50%, on a 12 h light dark cycle, with ad libitum access to standard chow food and water, unless otherwise specified. Male mice were housed in cages containing bedding, nesting material and enrichment with up to five individual animals, except for cold and thermoneutral experiments which were undertaken in empty cages containing singly housed mice. Age matched WT and Klf3-/- male mice derived from Klf3+/- x Klf3+/- crosses were used for all animal studies. Diet ingredients (chow and Western diet) are specified in Table S1.

Wild animals

No wild animals were used in this study

Field-collected samples

No field-collected samples were used in this study

## Flow Cytometry

### Plots

Confirm that:

- [x] The axis labels state the marker and fluorochrome used (e.g. CD4-FITC).
- [x] The axis scales are clearly visible. Include numbers along axes only for bottom left plot of group (a 'group' is an analysis of identical markers).
- [x] All plots are contour plots with outliers or pseudocolor plots.
- [x] A numerical value for number of cells or percentage (with statistics) is provided.

### Methodology

Sample preparation

Subcut and gonadal AT and lung were minced with sterile scissors then digested with 1 mg/mL type II collagenase, 20 mg/mL BSA in Hank's buffered salt solution (with calcium and magnesium). Digestion took place at 37oC for 45 min, shaking. Spleens were homogenised in PBS using a syringe plunger. AT SVF, lung and spleen cells were filtered through a 40 uM sieve then centrifuged at 500 x g for 10 min at 4oC. Red blood cell lysis was performed to remove erythrocytes, by adding ddH2O directly to cells then promptly adding 10X PBS to regain isotonicity. Cells were centrifuged again then stained for flow cytometry.CD16/32 (Fc block) was added to all samples to reduce non-specific binding. Fluorescently-conjugated antibodies and viability stains were added to cells and incubated on ice then washed off before proceeding (see Methods and Table S3 for antibody details).

Instrument

For flow cytometry, the BD LSRFortessa and LSRFortessa X-20 were used. For FACS, the BD Influx or BD FACS Aria II was used.

Software

FlowJo version 10 was used to analyse data

Cell population abundance

Following sorting on BD Influx of eosinophils derived from subcut AT SVF (approx 4,000-8,000 cells per mouse), RNA was immediately isolated and quality control performed on the Agilent BioAnalyzer, to ensure RNA integrity and sufficient yield to proceed with microarrays.

Gating strategy

Immune cell populations were defined as live CD45+. Within this, eosinophils were defined as CD11b+ F4/80+ SiglecF+ SSChi; macrophages as CD11b+ CD64+ CD11c+ (M1) or CD206+ (M2); dendritic cells as CD11b+ CD64- CD11c+; B cells as CD11b- CD64- CD19+ CD3-; T cells as CD11b- CD64- CD19- CD3+ then CD4+ for CD4 T cells and CD4+ CD25+ for Tregs; ILC2s as Lineage markers negative (CD11b- CD64- CD3- CD19- CD4- CD11c- NK1.1- CD127+ CD25+ and ST2+/-. Gating strategies can be found in Figs S7 and S8. To determine positive and negative populations, compensation was performed using single-stained control beads (UltraComp eBeads) and fluorescence-minus-one (FMO) control tubes.

- [x] Tick this box to confirm that a figure exemplifying the gating strategy is provided in the Supplementary Information.
